# A Review of Transparent Conducting Films (TCFs): Prospective ITO and AZO Deposition Methods and Applications

**DOI:** 10.3390/nano14242013

**Published:** 2024-12-14

**Authors:** Jessica Patel, Razia Khan Sharme, Manuel A. Quijada, Mukti M. Rana

**Affiliations:** 1 Division of Physics, Engineering, Mathematics and Computer Sciences and Optical Science Center for Applied Research, Delaware State University, Dover, DE 19901, USA; jessica.patel@nasa.gov (J.P.); khansharme@gmail.com (R.K.S.); 2 NASA Goddard Space Flight Center, Greenbelt, MD 20771, USA; manuel.a.quijada@nasa.gov

**Keywords:** TCO, thin film, ITO, antireflection, transparent electrode, transparent conducting film, TCF, indium tin oxide

## Abstract

This study offers a comprehensive summary of the current states as well as potential future directions of transparent conducting oxides (TCOs), particularly tin-doped indium oxide (ITO), the most readily accessible TCO on the market. Solar cells, flat panel displays (FPDs), liquid crystal displays (LCDs), antireflection (AR) coatings for airbus windows, photovoltaic and optoelectronic devices, transparent p–n junction diodes, etc. are a few of the best uses for this material. Other conductive metals that show a lot of promise as substitutes for traditional conductive materials include copper, zinc oxide, aluminum, silver, gold, and tin. These metals are also utilized in AR coatings. The optimal deposition techniques for creating ITO films under the current conditions have been determined to be DC (direct current) and RF (radio frequency) MS (magnetron sputtering) deposition, both with and without the introduction of Ar gas. When producing most types of AR coatings, it is necessary to obtain thicknesses of at least 100 nm and minimum resistivities on the order of 10^−4^ Ω cm. For AR coatings, issues related to less-conductive materials than ITO have been considered.

## 1. Introduction

Dielectric substrates are commonly applied with conductive (or thin-film) coatings. A precisely calibrated technique that preserves the coating’s mechanical, optical, and electrical qualities is needed to deposit such a delicate coating [1]. Materials with both optical transmissivity and electrical conductivity are known as transparent conducting oxides, or TCOs. Thin-film technologies and optoelectrical devices, including transparent p–n junction diodes, light-emitting diodes (LEDs), solar cells, and flat panel displays (FPDs), are made possible in large part by this special combination [2]. TCOs typically exhibit significant bandgaps (~4 eV), high refractive indices (between 1.70 and 2.05), high densities (7.14 g/cm^3^), and relatively high melting temperatures (~1900 °C) [3]. ITO, a colorless TCF with the minimum sheet resistance, increased transparency in visible light, high chemical stability, as well as excellent adhesion to a variety of substrates, is the most widely used and commercially available TCO in the material world [4]. Various coating techniques have been used to deposit ITO thin films, including spin-coating, inkjet printing, pyrolysis, ion beam sputtering, e-beam evaporation, DC or RF magnetron sputtering, and spray deposition [4]. In optoelectronic devices, including solar cells, displays, and touchscreens, aluminum-doped zinc oxide (AZO) is another frequently utilized TCO because of its low resistance and high transmittance [5]. Because of its electrical and optical characteristics, it is a material that shows promise for energy-efficient windows and sensors. AZO has great potential in next-generation flexible electronics because of its environmental stability and mechanical flexibility [6]. Research on improving its performance through sophisticated doping strategies and deposition procedure optimization is still ongoing.

The applications and performance potentials of AZO and ITO in contemporary technologies have been greatly expanded by recent developments in their research and development. A recent research study indicates that the combination of magnetron sputtering and post-deposition annealing, as employed in hybrid deposition techniques, has substantially enhanced the structural stability and efficiency of indium tin oxide (ITO) [7]. These techniques have made it possible to fine-tune the carrier concentration and defect states, guaranteeing excellent transmittance and strong conductivity in the visible spectrum. Similarly, because of its abundance, affordability, and less-negative effects on the environment, AZO has become a feasible substitute [8]. Its optical and electrical characteristics have been further enhanced by innovations in co-doping with elements, such as Ga and rare earths, allowing for increased adaptability in flexible and high-temperature applications. Both materials are now being modified for particular use in photovoltaics, where ITO films permit transparent top contacts that lower shadowing losses, while AZO films have shown promise as back reflectors [9]. Furthermore, new opportunities for high-performance devices with improved light–matter interaction have been made possible by their integration with nanostructured materials, like quantum dots and nanowires. These advancements demonstrate how important ITO and AZO are to developing flexible device, energy, and optoelectronic technologies.

In light of recent advancements, researchers are currently focused on enhancing electrical conductivity. To achieve this goal, Sn has been doped with ITO, a material derived from indium oxide (In_2_O_3_) [10]. Materials with low refractive indexes, for instance, MgF_2_ (*n* = 1.38) and SiO_2_ (*n* = 1.46), are utilized as coatings for ITO films in anti-reflection (AR) coating designs to minimize undesired reflections at the surface of the optical elements. SiO_2_ and MgF_2_ are electrical insulators, though, which restricts their use in ITO films for display device applications [10]. Sputtering at increasing temperatures in a growing environment with an ideal proportion of O_2_ proved to boost the electrical conductivity and optical transparency of ITO films, which, in turn, increased the overall GaAs solar efficiency [11]. On the other hand, AZO is developed from zinc oxide (ZnO) doped with Al and has a great balance between electrical conductivity and optical transparency. It is widely used in touchscreens, displays, and solar cells because it is less expensive and more environmentally friendly than ITO [12]. Although sustaining electrical qualities can be difficult in such combinations, the optical performance is improved by the addition of multilayer coatings, including materials with comparable refractive indices [13]. Developments in deposition methods, like high-temperature sputtering and pulsed laser deposition, have improved AZO’s performance even more for next-generation electrical and energy devices.

Several deposition techniques have been employed to fabricate ZnO films, including radio frequency (RF) magnetron sputtering, direct current (DC) sputtering, chemical vapor deposition (CVD), pulsed laser deposition (PLD), and atomic layer deposition (ALD). The production of superior thin films, such as ITO and AZO, for cutting-edge optoelectronic applications, depends heavily on CVD, a flexible deposition technique [14]. It is becoming increasingly important in contemporary material synthesis because of its capacity to provide exceptional homogeneity, accurate control over thickness, and customized film characteristics under ideal circumstances. Developments in CVD methods, such as atomic-layer-deposition-assisted CVD and plasma-enhanced CVD, can further enhance these coatings’ structural, optical, and electrical properties [13]. A thorough analysis of these new technologies’ effects on the creation of transparent conductive films will be included to improve knowledge of them. Each of these methods has its own unique advantages and applications, but ALD stands out because of its ability to produce homogeneous and conformal thin films on complex three-dimensional surfaces [15]. Furthermore, it allows for precise control over the thickness of the films at the atomic level, enabling tailored material properties for specific applications. The versatile nature of ZnO lends itself to a multitude of uses, including piezoelectric transducers, sensors, electronic components, and optoelectronic devices. Although significant research has focused on the deposition of ZnO thin films, the low deposition rates associated with current technologies have hindered advancements in thick film deposition, which could extend to tens of micrometers [16]. Lastly, the patterning of ZnO structures can be achieved through both wet and dry etching methods. Each approach offers its own set of benefits and challenges, providing researchers and engineers with options to tailor the fabrication process to meet the specific needs of their applications [17].

The ongoing advancements in deposition techniques for ITO can be effectively utilized when employing physical vapor deposition methods on glass substrates [18]. ITO can be etched consistently and quickly. The films also have a long lifespan because they are highly stable [3]. ITO is a special candidate for display applications because of these properties. Specifically, the most significant influences on these materials’ performance are their electrical and optical properties. Giving non-conductive materials electrical conductivity is one of the main purposes of conductive coatings [19]. Many substrates, including plastics and glasses, are, by nature, non-conductive. By applying a conductive coating, these materials can be turned into conductive surfaces that permit electric current to flow and facilitate the operation of electronic circuits and devices. Transparent conductive coatings are a result of the growing need for touchscreens, displays, and other transparent electrical interfaces. ITO is widely employed by numerous TCO applications because of its transparency, exceptional electrical conductivity, and straightforward deposition onto substrates, such as plastic and glass. The need for low optical reflection losses necessitates the use of transparent conductors in photovoltaic cells, OLED lighting, and display technologies. In contrast, ZnO is a semiconductor material distinguished by its remarkable properties, which render it highly advantageous for a range of modern technological applications. With a bandgap of 3.3 electron volts (eV) at room temperature (300 K), ZnO demonstrates impressive operational efficiency under various conditions [20]. Additionally, its Young’s modulus ranges from 150 to 240 gigapascals (GPa) in thin-film configurations, indicating considerable mechanical strength [21]. ZnO also exhibits strong thermal and chemical stabilities, ensuring durability across diverse environmental contexts. Additionally, its compatibility with micro- and nanofabrication techniques positions ZnO as a promising candidate for integration into cutting-edge devices. One of the notable enhancements to ZnO’s performance is the introduction of aluminum doping, which significantly boosts its electrical conductivity, expanding its utility in electronic applications. This makes ZnO a compelling alternative to traditional silicon, particularly in the realm of micro/nanoelectromechanical systems (MEMS/NEMS), where miniaturization and efficiency are paramount [22].

ITO coatings are also frequently applied in various highly developed optoelectronic instruments, such as FPDs, solar cells, lasers, and sensors, because of their high transmittances and low resistivities [23]. This is because in the visible solar spectrum, ITO is a degenerate semiconductor with strong transmissivity and conductivity. Nonstoichiometric ITO can also result from oxygen vacancies, which give rise to the conductivity altogether with Sn donors. When utilized as an AR coating, this nonstoichiometric ITO displays suboptimal optical characteristics [24]. ITO has the potential to be a transparent top contact for solar cells, which might lower processing costs and eliminate shadowing losses [24]. Reducing optical losses would enhance a solar cell’s absorption capabilities, which are essential for achieving high efficiency [25]. Therefore, in order for these cells to collect light, they must have an extraordinary AR capacity. Pyramidal texturing with alkali etching is used in current SHJ solar cells to obtain sufficient light trapping and AR properties. TCO reduces reflectivity and boosts photon infusion from the sun’s spectrum into the device when applied to a textured surface [25]. ITO coatings are becoming more and more well-liked because of their superior transparency, high dielectric strength, resilience in challenging environments, and outstanding stability. In addition, the cost of the solar cell is reduced, and its stability and efficiency are enhanced by concealing the top of the TCO layer with a dielectric coating [25]. TCO coatings in silicon heterojunction (SHJ) solar cells allow for a reduction in TCO thickness without sacrificing lateral conductivity. It should be noted that a thinner TCO can lower the costly TCO’s material consumption in addition to lowering the dependency on light absorption from the TCO itself. Furthermore, recent research has revealed that the stacking of SiN_x_/SiO_x_ has the potential to strengthen SHJs’ resistance to moisture, which enhances their stability in hot and muggy conditions. Consequently, TCO layers promise reduced costs and enhance the stability of solar cells and modules. In silicon SHJ solar cells, ITO is also utilized for AR to reduce the reflectance losses from incident solar radiation [26]. Section 2 enumerates some of the fundamental properties of TCOs. However, AZO coatings have emerged as a critical component in advanced optoelectronic devices, including touchscreens, OLEDs, photovoltaic cells, and various sensors. Their appeal lies in their remarkable optical transparency combined with exceptional electrical conductivity. The process of aluminum doping significantly enhances the carrier concentration within AZO, a type of transparent conducting oxide, while maintaining its inherently low resistance. However, it is important to note that defects can occur during the deposition process, potentially leading to the formation of nonstoichiometric AZO [27]. Such irregularities can compromise the optical and electrical performances of the material. Despite this challenge, AZO presents a promising solution for enhancing the overall device efficiency. When utilized as a transparent electrode in solar cells, AZO can effectively improve light absorption, reduce production costs, and mitigate shadowing losses, making it an invaluable asset in the quest for more efficient energy conversion technologies [9].

Engaging in detailed discussions surrounding theoretical models and density functional theory (DFT) simulations is crucial for unraveling the intricate atomic-scale interactions that occur at the surfaces of ITO and AZO [28]. Through the lens of DFT, researchers can explore the impacts of tin doping and the presence of oxygen vacancies, which play a significant role in enhancing the optical and electrical conductivities of ITO [29]. Additionally, DFT studies can illuminate how aluminum doping affects the electronic structure and carrier concentration of AZO, providing a clearer understanding of these materials’ properties. By leveraging these advanced theoretical methods, scientists can gain valuable insights into the nature of bonding, the behavior of electronic states, and the dynamics of defects. This comprehensive understanding ultimately paves the way for optimizing ITO and AZO, enabling their effective use in cutting-edge optoelectronic applications.

For the aerospace and defense industries to increase performance, safety, and efficiency, optical coatings, especially AR coatings, are essential [30]. By meeting the destructive interference criterion at a specific wavelength, these coatings increase the transmittance of thin films, reducing reflection and raising the corresponding transmittance [31]. Accurate data collection and navigation are made possible by enhanced signal-to-noise ratios and increased sensor sensitivity brought about by optimized light transmission [30]. Coatings are employed to make cameras and sensors more sensitive in space research (where light is scarce), enabling scientists to record far-off celestial occurrences with previously unheard-of clarity. Among the well-known AR coatings, AZO was developed specifically for NASA’s Hubble Space Telescope (HST) to minimize reflections from its instrument and optimize light capture. Such coatings have played a crucial role in producing amazing pictures of celestial occurrences and far-off galaxies [30]. Aerospace applications include stealth and radar technologies, coatings for temperature control on spacecrafts, AR coatings for aircraft glass, and improvements to sensor and imaging systems. Launched in 2020, the mission of the European Space Agency to solar orbit uses cutting-edge thermal management coatings to shield the spacecraft’s solar panels and equipment from sharp temperature swings as it gets closer to the sun. These coatings enable the spacecraft to collect important data about our star while withstanding the harsh solar radiation [30].

In this review article, we provide an overview of ITO films made with various deposition techniques, along with a thorough analysis of the literature. Apart from the deposition method with a few optimized parameters, a range of optical and electrical characterizations have been compared, including resistivity measurements, surface morphologies, x-ray diffractometry, UV-VIS-NIR spectrometry, mobility and charge carrier concentrations against temperature, and optical bandgap energies. This research also reviews, compares, and analyzes the research of authors who report on maximizing optical transmittance and decreasing resistivity concurrently [2,3,4].

## 2. Properties of TCFs

Enhancing the performance of current materials requires a thorough understanding of their basic characteristics. Furthermore, these discoveries have significant scientific implications for the creation and synthesis of novel TCOs [3]. TCOs are the primary components of optoelectronic and photonic devices and, as such, serve as a substrate/electrode or a catalyst, depending on the need for an efficient device. The TCO family includes metal oxides as follows: fluorine-doped tin oxide (FTO), pure zinc oxide (ZnO), aluminum-doped zinc oxide (AZO), and indium tin oxide (ITO). They are transparent between 350 and >800 nm in wavelength, and they exhibit a broad bandgap energy of around 3.4 eV. These TCOs can be fabricated into thin films with resistivities ranging from 1 to 5 × 10^−4^ Ω cm. ITO exhibits low resistance in the visible region and about 85% transparency, which make it an attractive candidate. Compared to AZO, ITO thin films are more readily formed utilizing sputtering deposition processes. TCOs can be amorphous or polycrystalline, and they are frequently doped with elements, like Cl, F, Sb, Ti, and Mo [32].

### 2.1. Chemical Durability

A TCO’s ease of etching is inversely correlated with its resistance to corrosive chemical conditions [33]. The most resistant is SnO_2_, but ZnO is easily damaged by bases or acids. Silver should only be used in hermetically sealed applications because it tarnishes easily in the presence of air and moisture. Therefore, the application of these metal oxides is limited because of their chemical instability in acidic and basic environments, which are corrosive scenarios [1]. Although oxide coatings are easily scratched, they are not affected by the majority of commercial solvents, including mineral spirits, xylene, naphtha, acetone, methyl ethyl ketone, and toluene. SnO_2_ is challenging to chemically etch, and this needs to be considered while developing innovative TCOs in the future.

### 2.2. Toxicity

Manufacturers of targets are particularly aware of toxicity concerns when it comes to metal targets. Toxic materials are among those employed in TCOs [1]. The requirements to safeguard employees and stop hazardous materials from escaping into the environment drive up the cost of handling them. In addition to extra encapsulation during use, facilities for recycling when a product’s lifespan is over might be required. The elements’ relative toxicities often rise in the following order: Cd > Ag > In > Sn > Zn [33]. Because the compounds of cadmium cause cancer, they are strictly regulated. For metallic silver or its inorganic salts to become poisonous, high dosages taken orally over an extended period of time are usually necessary. Only limited observations in humans and laboratory animal tests have been used to report on their toxicity.

### 2.3. Criteria for Choosing Transparent Conductors

When selecting TCOs, there are two primary indicators: First, there is high visible-light transmittance, and second, there is high electrical conductivity [34]. Low cost, high conductivity, reflectivity, and a surface area with the ideal thickness are the ideal characteristics of TCOs [35]. Transparent, electrical TCFs have been studied for a variety of semiconducting oxides, such as Sn, In, Zn, and Cd, as well as metal nitrides, such as Ag, Au, and Ti [33]. Additional factors that could influence the choice of the TCF material for a given application include the thickness; deposition temperature; toxicity; homogeneity; etchability; conductivity; plasma wavelength; work function; physical, chemical, and thermal endurances; as well as cost. TCOs can exhibit luminescence, high electron mobilities, and semiconductor and piezoelectric activities, and they have comparatively high melting temperatures. They also transmit at visible wavelengths [33]. Because they can both reflect and absorb infrared wavelengths, they are also efficient heat reflectors with UV protection. This will help to determine the best transparent conducting material. Although different transparent conductors (TCs) work well in different applications, there is not a single concrete answer that fits all situations. Additionally, the preparation procedure and, consequently, the material choice may be limited by a specific application. Different decisions are taken depending on which material quality is more important [33]. A summary of some of the key factors influencing the selection of a transparent conducting material is provided in Table 1. This chart makes it clear that there are a variety of purposes for TCs and that no single material is best suited for every situation. Different selections are made depending on which material quality is the most important.

### 2.4. Production Costs

The increasing need for compact, lightweight, and portable gadgets is driving up the rate of manufacturing of electrical and optoelectronic devices. As a result, there will eventually be a need to replace the heavy, bulky, fragile, conductive, and transparent materials that are currently in use [1]. Two primary factors determine the cost for TCOs: Global demand for certain applications, including solar batteries and flat panel displays, is directly driving up the prices of TCO glasses [36]. On the other hand, the largest TCOs in the glass industry are associated with coating types, like ITO- or AZO-coated glasses. The cost of producing a TCF material is determined by the prices of the processing of the thin layer as well as its necessary raw components. In general, the following order of raw material costs increases: Cd < Zn < Ti < Sn < Ag < In [33]. As a result of mining ores for other metals, like lead and zinc, indium is the most costly and rare that is mined, according to this ranking [33].

### 2.5. Cost-Effective Deposition Technique

The following is a typical order in which the expenses of the deposition technologies increase: pulsed laser deposition > magnetron sputtering > vacuum evaporation > atmospheric-pressure CVD > sol–gel [33]. The least expensive product produced using each method was considered while estimating this ranking. The process speed has a significant impact on costs. Compared to atmospheric-pressure CVD, low-pressure CVD requires more expensive equipment [33]. Graphite or TCO covered with platinum is typically utilized as a counter electrode [34]. MOs and metal chalcogenides are being employed as substitutes for pricey platinum lately. Moreover, among all the other deposition techniques, chemical solution deposition (CSD), such as dip- or spin-coating, is a popular method for quickly and affordably creating ITO thin films [35].

## 3. Deposition Techniques with Various Optimized Parameters

The authors have researched and used a variety of deposition techniques to produce high-quality ITO films. The magnetron sputtering process, which needs high temperatures and low pressures, is the most widely used technology for the deposition of TCO materials. In contrast to other transparent conductive metal oxides, ITO has a lower melting temperature than that required for deposition. Furthermore, ITO may be etched more readily than AZO if a clear conductive coating pattern is required. Furthermore, the sputtering method, which is frequently used to produce ITO thin films, is a simpler process than AZO deposition methods.

### 3.1. Magnetron Sputtering

Even with the wide range of uses for traditional transparent conductive metal oxides, there was a need to swap them out for more effective alternatives. Examples of traditional transparent conductive metal oxides utilized in photovoltaic and optoelectronic devices, as transparent conductive electrodes, are ITO and AZO, which have strong electrical conductivity and optical transparency. Among the various metal oxides, ITO provides very low resistance and a transparency of 85% [1]. Hu et al. produced ITO thin films using RF sputtering on glass substrates [2]. The target in this work was a ceramic with a thickness of 6 mm, In_2_O_3_-SnO_2_ (90:10 wt.%). To remove the surface oxide layer, a steady power of 120 W was applied for 0.5 h at a 50 sccm (standard cubic centimeters per minute) flow rate in an argon environment of 1.0 × 10^−2^ mbar. The substrate’s temperature was maintained below 50 °C. After the deposition, all the thin samples were annealed for approximately two hours at temperatures ranging from 200 to 500 °C in 100 °C intervals in air on a tube’s surface [2].

Dekkers used deposition targets made of commercially available INO and ITO [3]. In addition to pure In_2_O_3_, these targets’ stoichiometries were 2 wt.%, 5 wt.%, and 10 wt.% SnO_2_-doped In_2_O_3_, or ITO 2%, ITO 5%, and ITO 10%, respectively [3]. In the various experiments, the film thickness varied between 125 and 500 nm. Based on this research, it has been demonstrated that during thin film formation, the pressure of the oxygen and temperature of the substrate had the greatest effects on the electrical characteristics of ITO films [3].

Ali et al. discuss ITO films made using RF magnetron sputtering on glass substrates at 27 °C combined with Si substrates to investigate the deposited ITO thin films’ visible-light transmission qualities [37]. The films had a 40 nm thickness. In a pure Ar environment, the ITO target containing In_2_O_3_:SnO_2_ (90:10 wt.%) was maintained at a base vacuum pressure of 2.0 × 10^−5^ mbar [37]. As the plasma gas was sputtering, argon (Ar) gas was introduced to the chamber. RF powers of 50 and 75 W were maintained, while the operating pressure of the chamber was adjusted between 0.5 and 10 mTorr. The films had a 500 nm thickness. After the ITO films were deposited, the samples underwent post-annealing at temperatures ranging from 500 to 700 °C. Ahmed et al. reported a room temperature RF sputtering technique used to create 200 nm thick ITO films on glass substrates [22], and 90% In_2_O_3_ and 10% SnO_2_ were present in the sputtering target. The previous pressure was kept constant at about 10^−7^ mbar. A 200 nm thickness was attained by synthesizing the ITO thin films at 100 W of RF power. For one hour, the annealing procedure was carried out in ambient air at different temperatures [38].

Tipparach et al. used an Edward 350 sputtering system with a planar magnetron sputter gun that had a maximum power of 1000 W to create ITO films on glass substrates [39]. Obtaining high-quality ITO films requires careful attention to cleaning details. The substrates, which are microscope slides made of soda–lime silicate, are cleaned using ultrasonic waves. They are first cleaned using a liquid detergent solution for twenty minutes, then rinsed twice in deionized water, and then cleaned once more using ultrasonic waves in warm deionized water at a temperature of about 80 °C. Lastly, they are rinsed in flowing DI water and blown dry in nitrogen gas. The method used for sputtering was as follows: The In_2_O_3_ (90 wt.%) and SnO_2_ (10 wt.%) powders were completely ground, fired for five hours at 1000 °C in air, and heat-treated for five hours at 800 °C in an Ar environment. The resulting powder was reground and then formed into a cylindrical pellet with a diameter of 7.6 cm and a thickness of 0.32 cm. After the switch was switched off, the pellet spontaneously cooled to ambient temperature after being annealed at 800 °C for five hours in an Ar environment. The pellet served as the sputtering target.

Gwamuri et al. produced ITO films on glass substrates and (100) prime Si substrates with thermally generated oxide by utilizing a 99.99% pressed ITO (In_2_O_3_:SnO_2_: 90:10 wt.%) target [40]. The sputtering chamber’s pressure was kept at 7.5 × 10^−3^ Torr after it was started at a low (10^−7^ Torr) base pressure. The substrates and targets were maintained at a constant distance of 75 mm. The standard protocol called for pre-sputter cleaning of the target at 150 W, while film sputter deposition was carried out at 100 W. The oxygen gas flow was adjusted to 0, 0.4, and 1.0 sccm while maintaining a sputter rate of 8–12 nm/min, while the argon gas flow rate was kept constant at 10 sccm. It was observed that the sputter rate decreased as the oxygen flow rate rose. After deposition, ITO thin films were air-cooled and annealed for 30 min at 300 °C. Employing a standard HCl chemical etchant mixture, HNO_3_:H_2_O at a volumetric ratio of 1:1:5, ITO/Si films were etched. The Si/SiO_2_ films were etched at a low, regulated rate because all the etching was performed at room temperature. Following a thorough cleaning with DI water, the engraved samples were dried in a nitrogen atmosphere.

Tien et al. used DC magnetron reactive sputtering to create ITO thin films [41]. A mechanical pump supported a turbo molecular pump in the vacuum system. An In–10 wt.% Sn alloy disk measuring three inches was employed as a sputtering target. The distance between the target and substrates was 80 mm. To remove the adsorbed gas from the surface of the ITO target, the target was pre-sputtered for roughly ten minutes with a shutter covering it. The flow rates of an Ar and O_2_ mixed gas were separately controlled using mass flow controllers. This gas served as the operating gas. A DC discharge was maintained by a power supply operating in constant-current mode at 150 mA. Because the constant-current mode can lessen arcing and spikes, the coating process’s plasma conditions are more stable. The system’s basal pressure was 2.7 × 10^−4^ Pa. Ar and O_2_ were added close to the target and substrate, respectively, to reduce the oxidation of the target. The oxygen–reactive gas flow rates were adjusted between 10 and 50 sccm. Distinct oxygen flow rates cause distinct characteristics in the ITO thin films’ deposition. As coating substrates, both BK7 glass and Si wafers were utilized; the former was employed to gauge the ITO thin films’ transmittance and residual stress, while the latter was used to measure other parameters. During the film deposition process, the coated substrates were assumed to be at the ambient temperature because they had not been heated [41]. The ITO thin films’ surface roughness was measured using a microscopic interferometer, and residual stress was assessed using a homemade Twyman–Green interferometer.

#### Minimum Temperature for Deposition

To produce the necessary qualities in the TCs, the substrate temperature generally needs to be kept high enough while the TCs are deposited on it. The following sequence of increasing temperatures is typically needed: Ag or ITO < ZnO < SnO_2_ < Cd_2_SnO_4_ [33]. Therefore, for deposition on heat-sensitive substrates, like plastics, silver or ITO may be chosen, but cadmium stannate needs very refractory substrates to acquire its optimal features.

### 3.2. Electron-Beam Evaporation

**Figure 1:**
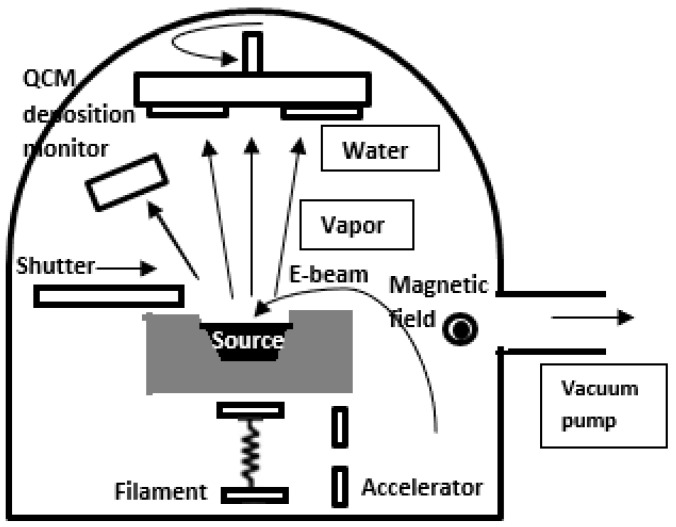
Schematic diagram of the e-beam evaporation system used to deposit ITO films. Reproduced from with permission from the copyright clearance center.

To create ITO thin films on glass substrates, Pokaipisit et al. used ion-assisted electron-beam evaporation (Denton DVB SJ26C, Moorestown, NJ, USA), as seen in Figure 1 [42]. ITO was used as the evaporation source material. Pure oxygen gas (99.99%) was delivered to the deposition chamber during the deposition procedure. At roughly 6 × 10^−5^ mbar, the pressure was maintained during the evaporation process. During the deposition process, a QCM (quartz crystal thickness monitor) was used to measure the thickness of the ITO thin films. For every film, the ITO thin film’s thickness and evaporation rate were kept constant at 120 nm and 2 Å/s, respectively. Throughout the deposition procedure, the substrate temperature was maintained at 150 °C using the radiation from quartz lamps. The revolving substrate holder and the electron-beam evaporation source were 60 cm apart. The oxygen flow rates were set up using an End-Hall ion source at 8, 10, 12, and 14 sccm flow rates, with a mass flow controller (MKS) serving as a monitor because it was believed to be a changeable parameter.

Menon et al. used a 12-inch vacuum-coating apparatus with an e-beam cannon to evaporate ITO thin coatings [43]. First, 99.99% tin oxide powder and spectroscopically pure indium oxide powder were completely mixed at different weight percentages using a pestle and mortar. In a furnace, the mixture was sintered at 800 °C for six hours. Pellets made from the combination were employed as the source materials for evaporation. Following the evacuation of the vacuum chamber, a base pressure of 1 × 10^−6^ mbar was attained. There was just pure oxygen within the chamber. By altering the oxygen needle valve, a consistent chamber pressure of 5 × 10^−5^ mbar was achieved. Glass slides were utilized as the substrates. For evaporation, three conditions had to be met: (1) an e-beam current of 10^−5^ mA, (2) an acceleration voltage of 6 kV, and (3) a vacuum of 5 × 10^−5^ mbar. The amount of evaporation was changed between 13 and 15 nm/min [43].

### 3.3. Dip-Coating

Because the wet coating process requires less capital and basic infrastructure, it is an advantageous way to produce ITO thin films at a lower cost [9]. ITO precursor films were deposited by Ito et al. using a dip-coater on Si (100) and silica glass (20 mm × 40 mm × 0.85 mm) substrates at substrate withdrawal rates of 0.050–1.0 cm min^−1^ [44]. To maintain the coating temperature or the combined temperature of the substrates, coating solutions, and environment at 60 °C, the dip-coating procedure was carried out in a thermostatic oven. The substrates and coating solutions were heated in the thermostatic oven for 30 min at 60 °C prior to dip-coating. Precursor films made on silica glass substrates were heated for 10–60 min at 500–800 °C while maintaining a 0.5 L min^−1^ N_2_ gas flow to create ITO films. After that, the precursor films were put directly into an electrical furnace, in a tube that was kept at the proper temperature. The films obtained from one to six cycles of dip-coating and heating were employed in the measurements. Cu Kα radiation (λ = 1.54056 Å) in an XRD diffractometer was used to study the crystalline phases of the precursor and heat-treated films.

### 3.4. Spin-Coating

By the spin-coating process, ITO films were deposited utilizing two distinct precursor systems, as reported by Solieman et al. [45]. The first was a traditional sol made by dissolving tin acetate and indium nitrate in ethylene glycol. Following this step, the mixture was diluted with ethanol. The coating had five layers, totaling 60 nm in thickness. A crystalline ITO nanoparticle suspension that had been redispersed in ethanol to a primary size of about 25 nm, was used in the second one, which was created using a patented procedure. There was just one 640 nm thick layer of coating. The two coatings were sintered for 30 min at 550 °C and then they were annealed for 30 min at 350 °C in forming gas (N_2_/H_2_: 92/8). Using a Cary 5E spectrophotometer for UV-VIS-NIR and a Fourier-transform infrared (FTIR) spectrometer (Bruker IFS 66v, Billerica, MA, USA) for IR, the reflectance and transmittance spectra of the coatings and the glass substrates were measured in the 250 nm–20 μm wavelength range. The electron carrier density (n_e_), resistivity (ρ), and carrier mobility (μ), the three dc electrical properties, were ascertained using the four-point, van der Pauw and Hall procedures (MMR Technologies, San Jose, CA, USA). A surface profiler, model Tencor P-10 (KLA, Milpitas, CA, USA), was used to measure the coatings’ thickness [45]. High transparency, high conductivity, and low sheet resistance are essential for ITO films, which will improve the performance of the antireflection layer (of solar cells) [46].

### 3.5. Inkjet Printing

Pan et al. prepared a thin-film ITO precursor using inkjet printing on a 20 × 20 mm^2^ glass slide. They utilized a commercial flatbed printer that was adapted to use an Epson L805 [47]. The thin-film ITO precursor was then sintered for three minutes at 300 °C on a hotplate that had been warmed. About 58 nm thick ITO thin films can be produced using a single printing/sintering process. As a result, four printing/sintering cycles were repeated to create the 230 nm thick ITO thin film. Lastly, the ITO was heated for 30 min at 350–550 °C in a furnace with forming gas (5% H_2_ + 95% N_2_).

### 3.6. Pulsed Laser Deposition

Socol et al. used the PLD method to deposit ITO films on nanopatterned and flat glass substrates [48]. A solid ITO target was ablated using 7000 laser pulses. The laser beam was focused at a 45° incidence angle on the target surface using a 300 mm focal length MgF_2_ lens that was placed outside the deposition chamber. In order to reduce the local damage, the target was circulated during the deposition process, and the distance between the substrate and the target was fixed at 5 cm. In the deposition chamber, the oxygen was pumped to a pressure of 1.5 Pa, leaving a residual pressure of 10^−4^ Pa. The PO_2_ (oxygen pressure) was chosen because ITO films with low resistivity values can be produced at 27 °C at a pressure value of 1.5 Pa [48]. The structured nanostructures were spared deterioration using a modest laser fluency of 1.2 J/cm^2^ and by maintaining the substrates at room temperature.

## 4. Optoelectrical Properties of ITO Films

After ITO thin films are deposited, a variety of optical instruments, including x-ray diffractometers, atomic force microscopes (AFMs), UV/VIS spectrophotometers, scanning electron microscopes (SEMs), and four-point probe stations, can be utilized to assess the structural, electrical, and optical properties.

**Figure 2:**
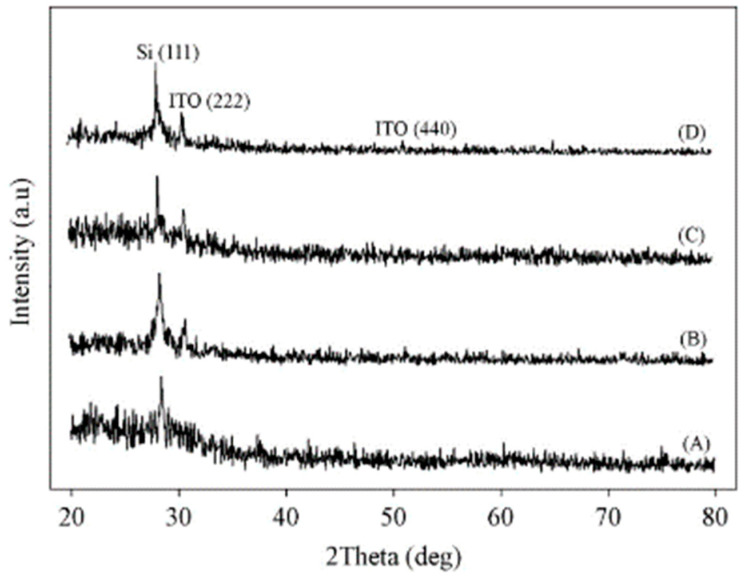
The XRD patterns for the phase analysis of the ITO on Si films (**A**) as deposited and post-annealed at (**B**) 500 °C, (**C**) 600 °C, and (**D**) 700 °C. Reproduced from [37] with permission from the copyright clearance center.

Ali et al. studied the effects of post-annealing after generating ITO thin films using RF sputtering on Si substrates [37]. The ITO thin films’ crystallization properties were examined (Figure 2). The as-deposited ITO was originally amorphous, as shown in Figure 2A. According to XRD patterns B, C, and D, a crystalline peak of ITO was observed at about 30.75° during the samples’ post-annealing procedure, which involved increasing the temperature from 500 °C to 700 °C (Figure 2B–D). This peak is associated with the indium oxide’s cubic bixbyite structure’s (222) reflections. Strong crystallization was seen in ITO thin films that had undergone post-annealing at 500–700 °C.

**Figure 3:**
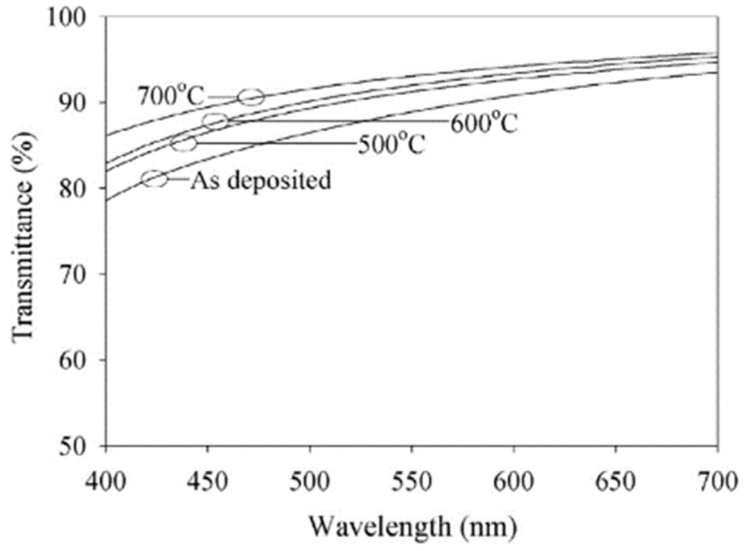
Transmittance spectra for the ITO thin films as deposited and after annealing are displayed. Reproduced from [37] with permission from the copyright clearance center.

Following post-annealing, the samples were optically measured in the spectral range of 400–700 nm on glass substrates. The transmittance spectra are displayed in Figure 3 for the as-deposited and post-annealed samples for temperatures ranging from 500 to 700 °C [37]. The transmittance of the ITO samples gradually increases with increasing wavelength. Following post-annealing at 500–700 °C, the transmittance of the ITO samples gradually increases to more than 85%. The ITO thin films’ transmittance significantly rose above 90% when the post-annealing temperature was increased to 700 °C. ITO’s transmittances are 90.4%, 92.6%, and 94.8% at 470 nm, 530 nm, and 630 nm, in that order. These findings demonstrate a notable increase in the optical transmittance through the ITO.

**Figure 4:**
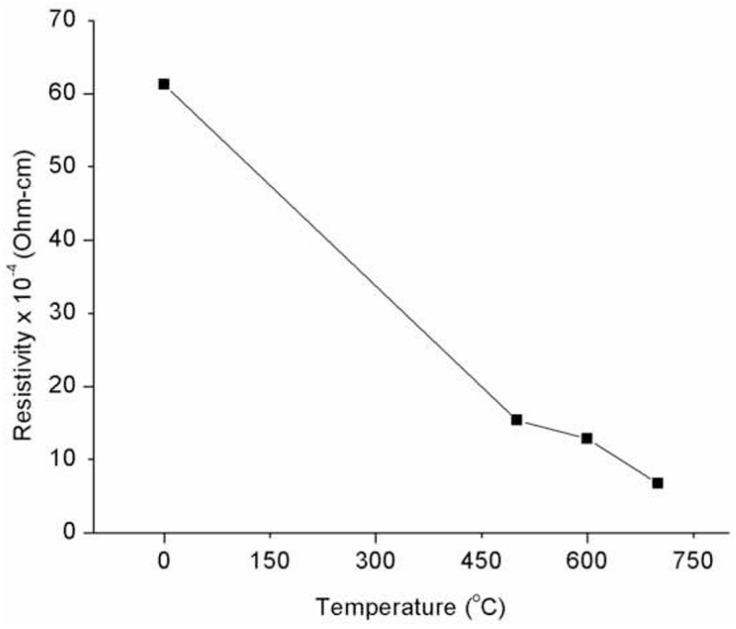
ITO’s electrical resistivity was measured, and its relationship to the post-annealing temperature was determined. Reproduced from [37] with permission from the copyright clearance center.

Using a van der Pauw test arrangement, a Hall system of model Accent (Melbourne, FL, USA) HL5500PC was used to analyze the electrical resistivity [37]. In Figure 4, the electrical resistivities of the ITO thin films, both as deposited and after annealing, are examined using the Hall effect setup. The resistivity of the latter significantly drops by nearly an order of magnitude, when comparing the post-annealed samples to the as-deposited samples. Post-annealed and as-deposited samples showed resistivities of 6.68 × 10^−4^ Ω cm and 61.23 × 10^−4^ Ω cm, respectively. As seen in Figure 4, the ITO’s resistivity increased, particularly at the 700 °C post-annealing temperature, when the lowest resistivity of 6.68 × 10^−4^ Ω cm was attained [37].

**Figure 5:**
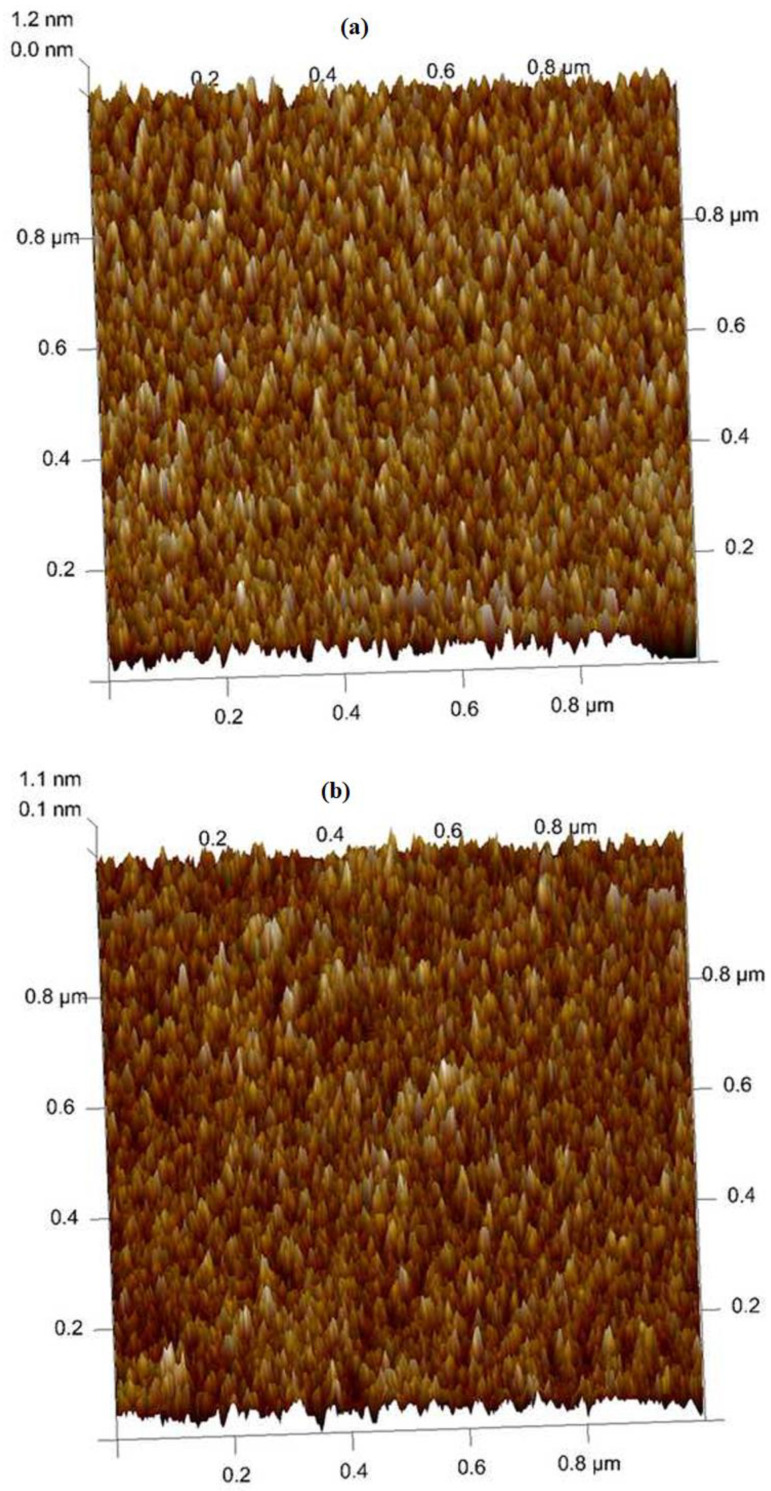

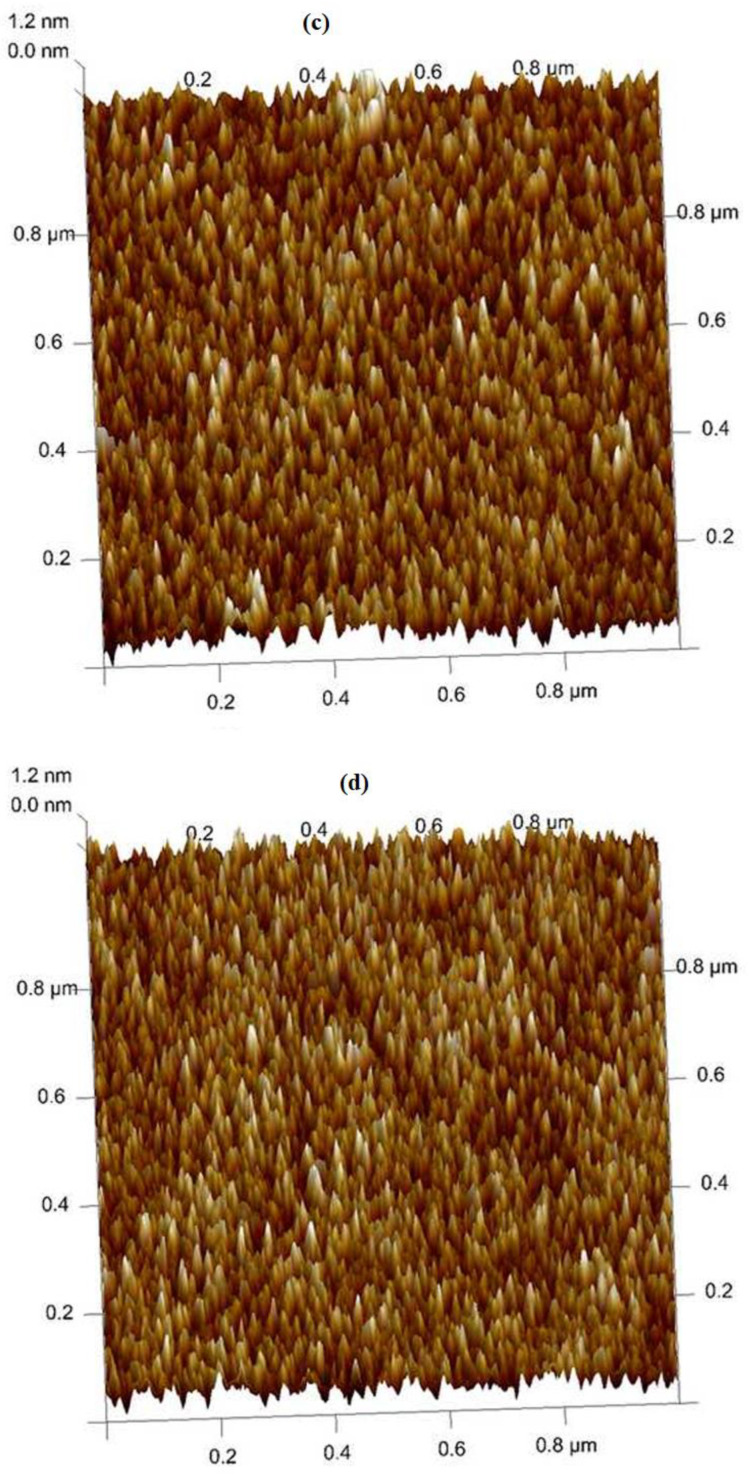
At three distinct post-annealing temperatures, surface roughness was measured for ITO thin films on Si using 3D AFM: (**a**) as deposited, (**b**) 500 °C, (**c**) 600 °C, and (**d**) 700 °C. Reproduced from [37] with permission from the copyright clearance center.

Figure 5 displays the surface properties of ITO following post-annealing at various temperatures [37]. AFM images were scanned over a 1.0 × 1.0 μm^2^ surface. When comparing the post-annealed samples to the as-deposited sample, it is proved that the surface roughness has decreased. According to measurements made using NanoScope Analysis software, the ITO’s grain sizes fall between 13 and 18 nm. This measurement and the computed outcomes from the XRD data are nearly identical. Additional measurements of the root-mean-square (Rq) surface roughness reveal that the surface morphology smoothens with increasing post-annealing temperature.

Additionally, the post-annealed ITO thin films’ optical and electrical properties are enhanced by a smoother thin-film surface. As a result, the annealing process enhanced the ITO films’ surface shape and structure while also raising their electrical resistivity [37]. In comparison to the as-deposited sample, the post-annealed samples’ structural and surface morphologies (Figure 5a–d) and optical (Figure 3) and electrical features (Figure 4) were greatly enhanced by post-annealing up to 700 °C. A rise in the substrate temperature causes the developed films’ grains to become bigger. This raises the conductivity of the ITO films and decreases the amount of grain boundary scattering.

Table 2 displays the transmission and resistivity values that have been reported thus far, together with the typical TCO thin-film materials and appropriate dopants. It should be mentioned that the ITO used in the AR coatings was created using the DC or RF magnetron sputtering process on amorphous substrates, like glass or Si [49]. Furthermore, in order to fabricate the majority of the AR coatings, deposition at a substrate temperature below 200 °C is necessary, as is the possibility of thicknesses from roughly 50 to 200 nm and generating low electrical resistivities.

Table 2 illustrates how difficult it is to employ TCO materials based on cadmium oxide and titanium oxide in practice because of the need for high-temperature heat treatment and harmfulness, respectively. To achieve a lower resistivity of around 10^−4^ Ω cm in TiO_2_-based TCO thin films and TiO_2_, high-temperature deposition, heat treatment above approximately 300 °C, and epitaxial growth on a single crystal substrate are required [50,51,52].

**Figure 6:**
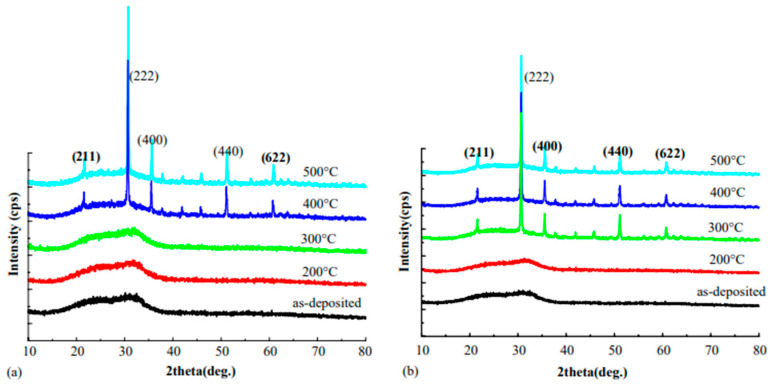
ITO films’ XRD patterns after being deposited and heated to various temperatures (**a**) in air and (**b**) under vacuum. Reproduced from [2] with permission from the copyright clearance center.

The XRD patterns of the ITO films are shown in Figure 6, following their deposition and annealing at different temperatures, both in air and under vacuum [2]. It is clear that the ITO films that are deposited without heating are amorphous. After annealing at a temperature above 400 °C in air or above 300 °C under vacuum, crystallization occurs. The majority of the annealed ITO thin films have a cubic structure, and the reflections of (211), (222), (400), (411), and (622) are represented by their diffraction peaks. Atoms and particles that were put on the substrate will move less freely at low substrate temperatures.

The amorphous films exhibit numerous structural flaws and a nonstoichiometric composition, as demonstrated in Figure 6 [2]. In order to create robust polycrystalline films, post-annealing can oxidize nonstoichiometric compositions, like In_2_O_3-x_ and SnO_2-x_, and rearrange atoms. The films may react with the free oxygen in the air in an environment. Thus, as the films anneal, the crystalline structure becomes more flawless, as observed by the films’ peak intensity.

**Figure 7:**
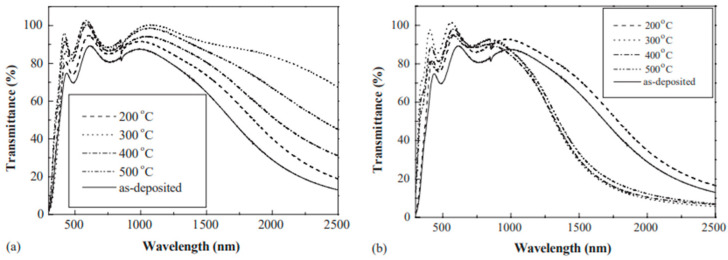
The transmittance spectra of the ITO thin films under diverse environmental conditions, including (**a**) in air and (**b**) under vacuum, at different temperatures during deposition and annealing. Reproduced from [2] with permission from the copyright clearance center.

Figure 7 displays the transmittance percentage (%T) spectra in both the visible and NIR bands for the ITO films that were deposited and annealed at varying temperatures either in air or under vacuum [2]. It demonstrates unequivocally how annealing improves a film’s transmittance in the visible-light spectrum. Adding an inert gas also prevents unintentional chemical reactions from deteriorating a sample. Therefore, a mass flow controller was used to maintain the Ar flow rates (99.999%) at a steady 50 sccm. High luminous transmittance can be obtained by annealing in an inert or an oxidizing environment, according to research by Hamberg and Granqvist [53].

The typical transmittance percentage (%T) of films in the visible-light spectrum is approximately 75% when they are not annealed, and it can reach over 90% when they are annealed at 400 °C in air or at 300 °C under vacuum (Figure 7). When the transmittance of the films reaches its maximum, the annealing temperature under vacuum is lower than that in air. Figure 7 illustrates how annealing affects crystallization and shows that the transmittance of amorphous films is significantly lower than that of crystalline films. We may observe that the films that were annealed in air have better transmittance in this range. When the film is annealed in air at 300 °C, its transmittance approaches its maximum. The transmittance of amorphous films is higher than that of crystalline films when they are annealed under vacuum [2].

The effects of the flow rate of the oxygen on the optical and electrical properties of ITO thin films were studied, as Table 3 demonstrates [42]. The sheet resistances (in Table 3) of the ITO thin films are shown, which were produced under the above-described circumstances utilizing different oxygen flow rates. The ITO thin films’ resistivity drops from 15.6 × 10^−4^ Ω cm to 7.2 × 10^−4^ Ω cm. Consequently, the highest bandgap (4.19 eV) and transmittance (84%) are seen in the ITO thin films formed at 12 sccm, indicating good optical and electrical features.

An effective estimate of the depths of the discrete profile deviation or average heights is the RMSSR parameter. The RMSSR and the ITO films’ surface morphology were examined with an AFM equipped with a tapping mode called NanoScope III. As we can see from Table 3, when the flow rate of the O_2_ increases from 8 to 12 sccm, the RMSSR climbs to 0.93 nm and decreases to 0.38 nm when the flow rate of the O_2_ reaches 14 sccm [42].

The sheet resistance (R_s_) was measured using the four-point probe method. The film resistivity (ρ) was calculated using the straightforward formula r = R_s_·d, where R_s_ is the sheet resistance, and d is the film thickness, and the assumption that the film thickness was uniform (Hamberg and Granqvist, 1986) [53]. The average of three measurements for every film was used to obtain the values of the resistivity and sheet resistance.

The transmittance spectral variation of the ITO thin films formed under identical conditions is listed in Table 3. The study [42] investigated that the ITO thin films exhibited a maximum optical transmittance of 84%, which they achieved at a flow rate of 12 sccm of oxygen. As the oxygen flow rate increased, the transmittance declined. The average optical transmittance of the ITO thin films at various flow rates of oxygen clearly affects the transmittance [42]. There is an increase in the optical transmittance below a 12 sccm oxygen flow rate. When the oxygen flow rate is increased appropriately, the optical transmittance (%T) can also increase. ITO thin films with a thickness of 120 nm exhibit the best optical transmittance at oxygen flow rates up to 12 sccm. An increase in oxygen’s flow rate above 12 sccm will result in a decrease in the optical transmittance.

Because sub-oxides (such as SnO_x_ and InO_x_) can be oxidized with a rising oxygen flow rate, ITO thin films have a higher transmittance. However, as confirmed by Hamberg and Granqvist (1986), grain boundaries and microcracks are examples of flaws where extra oxygen may be absorbed when the O_2_ flow rate exceeds the maximum [53]. Repetitive oxygen exposure can result in optical scattering and absorption. Moreover, Equation (1) was used to determine the optical bandgap (eV) values of ITO thin films from the transmittance spectra as follows:

(1) $$\alpha =d^{-1}\;ln\;(1/T)$$

In Equation (1), absorption coefficient, film thickness and transmittance are denoted by *α*, *d* and *T*, respectively.

The absorption increases with increasing optical energy and reaches its minimum at low energies, like the semiconductors’ absorption edge. Tauc’s equation, which is stated in Equation (2), can be used to represent the absorption coefficient for the directly allowed transition for the simple parabolic scheme.

(2) $$\alpha h\nu ={\left(h\nu -E_{g}\right)}^{1/2}$$

In Equation (2), the photon energy is symbolized as hν. Plotting (α*hν*)^2^ vs. *hν* and using the extrapolation approach will yield the optical bandgap (*E_g_*) of the ITO films. The value of (α*hν*)^2^ versus *hν* fluctuates for the ITO thin films made by Li et al. [42]. An increase in the oxygen flow rate from 8 to 12 sccm is shown in the study to correlate with an increase in the bandgap energy from 4.15 to 4.19 eV [42]. Because of their inverse relationship, the maximal optical transmission and electrical conductivity cannot be achieved simultaneously. As a result, a figure of merit (Φ) for contrasting TCO films with ITO has been created. According to Haacke’s (1976) definition, the figure of merit compares the performances of ITO thin films as Φ = T^10^/R_s_. The ITO films deposited at a 12 sccm O_2_ flow rate showed the highest value of the figure of merit, as displayed in Table 3.

**Figure 8:**
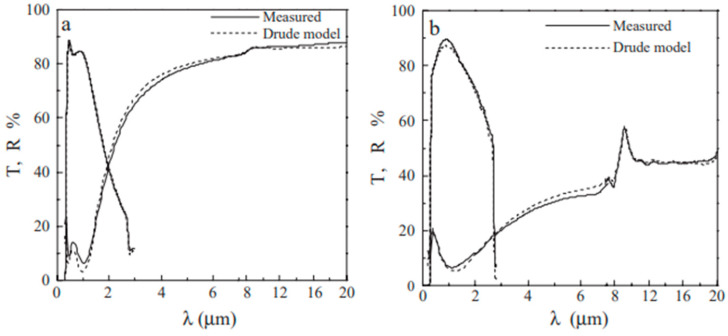
Transmittances (%T) and resistivities (%R) of (**a**) Asahi ITO film and (**b**) conventional sol–gel ITO film (were measured and fitted. Reproduced from [45] with permission from the copyright clearance center.

Figure 8 compares the electrical parameters that were obtained through modeling with those that were gained through experimentation. The Drude model has successfully fitted the optical data of an Asahi-glass-sputtered film with a resistivity of 1.9 × 10^−4^ Ω cm, a low porosity of 28%, and a fixed resistivity of 6.3 × 10^−4^ Ω cm, prepared using a sol–gel method from an ethylene glycol solution of Sn and In salts and sintered at 550 °C [45].

**Figure 9:**
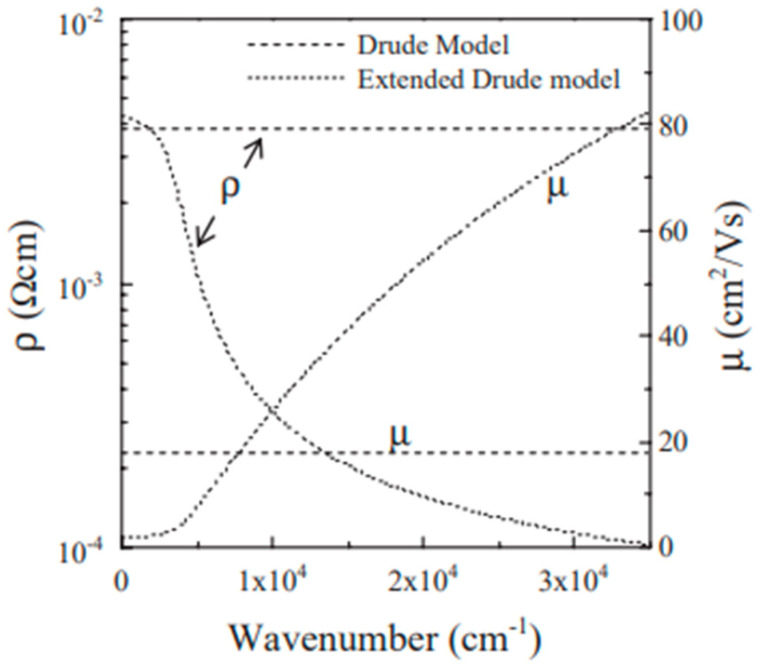
Electrical resistivity and mobility (μ) spectra were calculated from the fit of the optical data in Figure 10b. Reproduced from [45] with permission from the copyright clearance center.

Figure 9 displays the frequency dependence of the predicted electrical resistivity and charge carrier mobility derived using both methods [28]. This suggests that there is less interaction between the scattering centers and the electrons at high frequencies. The mobility similarly grows with increasing frequency continuously, as ρ = 1/n_e_eμ.

**Figure 10:**
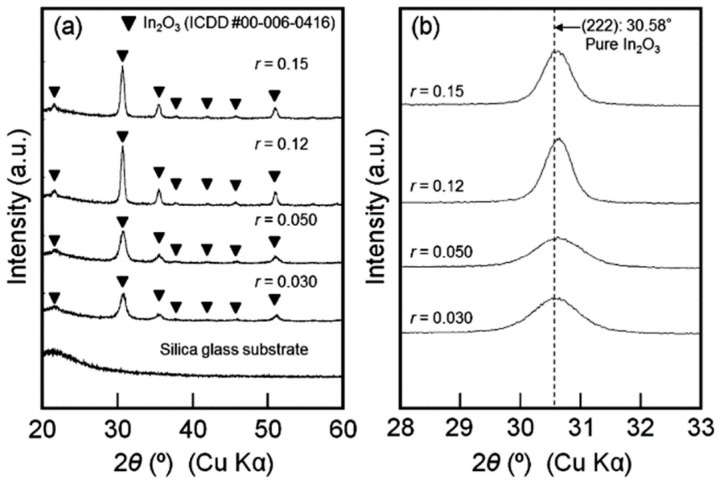
The heated ITO films’ XRD patterns, which were made from an aqueous solution with r = 0.030–0.15, are as follows: (**a**) from 20 to 60° and (**b**) from 28 to 33°. Reproduced from [44] with permission from the copyright clearance center.

Here, r was used to define the Sn/In molar ratio using the aqueous solutions. The XRD patterns of the heated films obtained at r = 0.030–0.15 are displayed in Figure 10. For every heat-treated film, the bixbyite In_2_O_3_ phase was found (Figure 10a) [44]. In comparison to the XRD peaks of the pure In_2_O_3_ phase, the XRD peaks of the ITO heated films of r = 0.030–0.15 were marginally displaced to higher angles (Figure 10b). This shows that Sn^4+^ ions have replaced In^3+^ ions. The bixbyite lattice may contract as a result of the substitution of Sn^4+^ ions because the ionic radius of Sn^4+^ is smaller than that of In^3+^.

**Figure 11:**
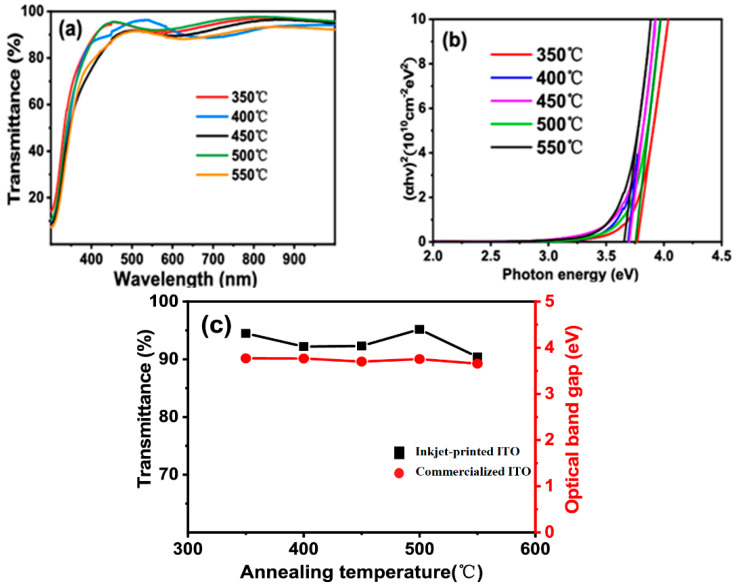
Optical transmittance (%T) spectra (**a**) and annealing-temperature-dependent optical transmittance and bandgap (**b**,**c**) of inkjet-printed ITO thin films annealed at various temperatures. Reproduced from [47] with permission from the copyright clearance center.

Metash (Shanghai, China) UV-5200 obtained UV-VIS-NIR transmittance spectra in the 300–1000 nm range [47] for the ITO thin films inkjet printed and annealed at various temperatures, and these spectra are displayed in Figure 11a. It was found that the ITO thin films’ annealing temperature and optical transmittance had a slight association. The high optical transmittance of the inkjet-printed ITO thin films is demonstrated by the fact that ITO thin films with varying annealing temperatures can attain over 90% transmittance on average in the 400–1000 nm wavelength range. Equation (1) is utilized to compute the absorbance coefficient (α) and absorbance (*A*) based on the optical transmittance (*T*). Next, we obtain the (αh*v*)^2^ vs. photon energy curve. Plots of (αh*v*)^2^ vs. photon energy for ITO thin films produced by inkjet printing and annealed at various temperatures are shown in Figure 11b [47]. By extending the linear portion of the curve, one may determine the optical bandgap of the ITO thin film produced by inkjet printing. The transmittances and optical bandgaps for ITO thin films produced by inkjet printing and annealed at various temperatures are displayed in Figure 11c. The ITO thin films’ optical bandgaps fall between 3.65 and 3.77 eV, which is comparable to those of ITO thin films that are sold commercially. Furthermore, the ITO thin film annealed at 500 °C reached the maximum transmittance of 95.2%. Consequently, in the 400–1000 nm range, the ITO thin film with a thickness of 230 nm shows a transmittance of 95.2%, a sheet resistance of 99 Ω/□, and resistivity of 2.28 × 10^−3^ Ω cm at the ideal annealing temperature of 500 °C.

**Figure 12:**
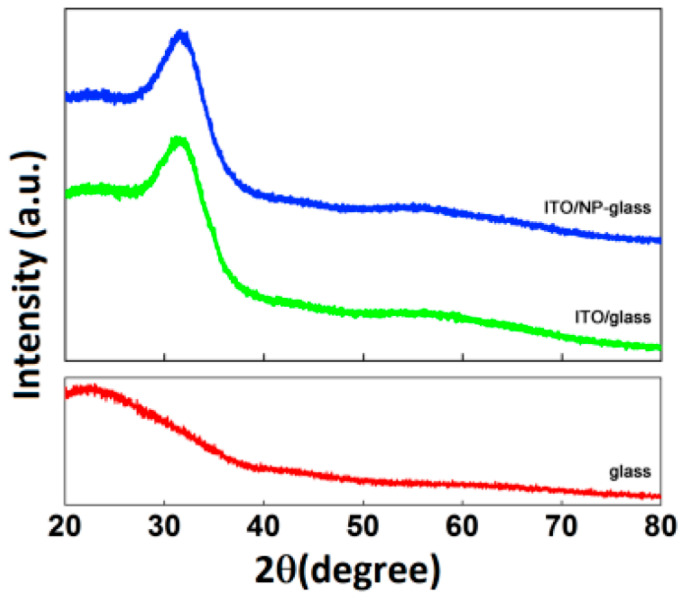
XRD patterns of glass, ITO/NP–glass samples, and ITO/glass. Reproduced from [48] with permission from the copyright clearance center.

The crystalline structure of the TCO films produced using PLD is significantly influenced by the substrate temperature and oxygen pressure [48]. Thus, XRD was used to examine the ITO/glass and ITO/NP–glass samples. Only a broad peak at around 31° is visible in the XRD pattern of the ITO thin films deposited on non-annealed substrates, as shown in Figure 12, which is consistent with a microcrystalline structure that has a favored (222) orientation [48]. It was observed that the ITO crystallinity is unaffected by the patterning technique.

Hall measurements (Table 4) were used to assess the electrical properties of the ITO films produced using PLD on nanopatterned and flat glass substrates. This demonstrated that the charge carriers are the electrons [48].

Because the TCO sheets generated an n-type semiconductor, the electrical measurements, irrespective of the substrate type, exhibited a low electrical resistivity of <2.8 × 10^−4^ Ω cm. On the other hand, ITO films placed on nanostructured glass showed an increase in the Hall mobility.

**Figure 13:**
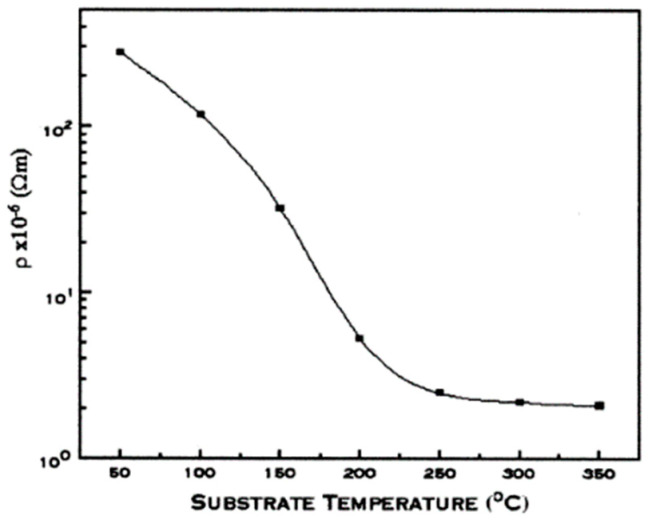
Variations in resistivity with substrate temperature for ITO films. Reproduced from [43] with permission from copyright clearance center.

Figure 13 displays the plot of the resistivity vs. the substrate temperature (Ts) for ITO thin films (In_2_O_3_/SnO_2_ = 80:20 wt.%) placed at various substrate temperatures. The film had a thickness of 150 nm [43]. There was a considerable decline in the resistance in the 50–250 °C temperature range. When the substrate temperature reached 350 °C, the lowest resistance measured was 3 × 10^−6^ Ω cm. The enhanced crystalline structure of these coatings may be the cause of the resistivity’s reduction with increasing substrate temperature.

## 5. Issues Associated with Substituting AZO

One of the current research hotspots is transparent conductive films, particularly indium tin oxide (ITO) films, an n-type degenerated semiconductor with a broad bandgap [54]. However, it becomes crucial to identify if a better substitute material exists. When employing AZO thin films instead of ITO thin films in AR coating applications, there are numerous problems that must be fixed. The two most important issues that still need to be resolved are the growth of thin-film preparation techniques appropriate for AR coating applications and the enhancement of thin-film stability in diverse conditions of thicknesses less than 100 nm [49]. It is thought that most of these issues can be resolved in the long run. For AR coating production processes, suitable thin-film preparation techniques must meet the following minimum requirements: Over a wide region, AZO thin films, about 50 nm thick, with a resistivity of around 10^−4^ Ω cm, must be created at a high deposition rate.

Furthermore, improving stability under various circumstances could also be essential. Although AZO thin films with resistivities on the order of 10^−5^ Ω cm have been reported to be produced using preparation techniques like PLD, the aforementioned characteristics most likely limit the possible preparation techniques for magnetron sputtering deposition [49]. Nevertheless, the issues associated with depositing AZO thin films and creating steadiness in thin films thinner than 50 nm need to be solved for the effective use of CVD technology. Therefore, the only suitable and practical preparative methods for using the AZO thin films in AR coating production processes are magnetron sputtering (MS) deposition techniques.

In a different case, magnetron sputtering at the ambient temperature was used to deposit the stack of AZO/Ag/AZO, which are transparent flexible electrodes, on (001) fluorphlogopite mica substrates (10 × 10 × 0.2 mm^3^) [55]. Using an ultrasonic bath, these substrates were successively submerged in the following solutions: ethanol, acetone, and distilled water for roughly five minutes each. Finally, dry nitrogen was blasted on the substrates. The working gas used was argon (99.99%), and the base pressure was kept at roughly 1 × 10^−4^ Pa. A target–substrate distance of 67 mm was chosen. The AZO layer was created using an AZO ceramic (99.99% pure, Al_2_O_3_:ZnO = 2:98 wt.%) with a working pressure of 0.55 Pa and pulsed sputtering with a pulse power of 85 W. The power and work pressure for the Ag layer’s direct current (DC) sputtering were 80 W and 0.48 Pa, respectively. The length of the sputtering process determined the thickness of each Ag and AZO layer. Following deposition, each sample was annealed for 30 min at 250–750 °C, or around 1 × 10^−4^ Pa [55].

**Figure 14:**
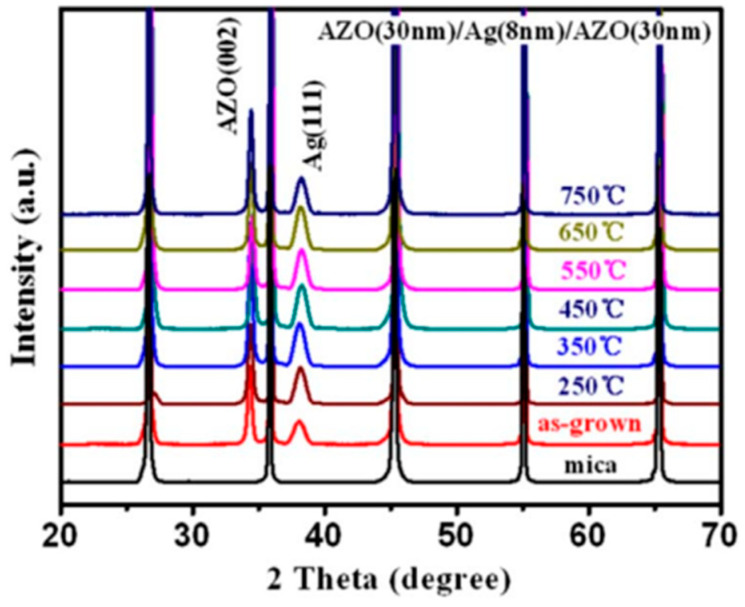
XRD patterns of the AZO/Ag/AZO multilayer stacks on mica sheets at different annealing temperatures. Reproduced from [38] with permission from the copyright clearance center.

As seen in Figure 14, the films’ crystallinity and orientation were assessed using XRD (DMAX1400, Rigaku, Tokyo, Japan) with Cu Kα radiation (λ = 0.154 nm) [55]. It displays the XRD patterns of the multilayer electrodes made of AZO, Ag with a thickness of 8 nm, and AZO on a mica sheet at different annealing temperatures. Regardless of the heating temperature, no extra diffraction peaks are visible when compared to the XRD pattern of the as-deposited material. Ag (111) peak’s diffraction intensity steadily rises with increasing annealing temperature, suggesting that heat treatment can enhance the film’s crystalline quality. This is because of the possibility that during annealing, the atoms’ increased thermal energy will cause them to diffuse and stack. Nonetheless, an overly high temperature would cause the Ag (111) peak’s strength to decrease.

**Figure 15:**
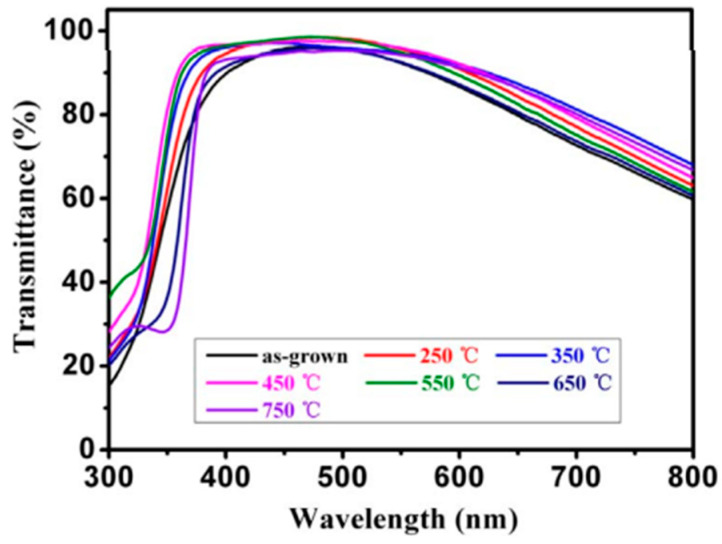
The AZO/Ag/AZO films’ optical transmittance (%T) spectra recorded at various annealing temperatures and for the as-deposited sample. Reproduced from [55] with permission from the copyright clearance center.

Using bare mica as a reference, optical transmittance (%T) was measured using a UV-VIS-NIR spectrophotometer (Solidspec-3700, Shimadzu, Kyoto, Japan) between 300 and 800 nm [55]. Figure 15 displays the transmittance spectra following heat treatment. In the visible spectral range of 400–800 nm, the average transmittance (%T) values of the AZO/Ag/AZO films were 83.1%, 86.8%, 88.2%, 87.7%, 85.8%, 83.6%, and 87.0% for the samples as deposited and post-annealed at different temperatures, respectively. It is clear that as the annealing temperature rises to 350 °C, the transmittance (%T) increases. This is because following the thermal annealing process, enhanced crystal quality causes a decrease in the optical absorbance at the grain boundary. Nevertheless, the optical transmittance (%T) of the AZO/Ag/AZO multilayer films somewhat decreases when the annealing temperature rises [55].

**Figure 16:**
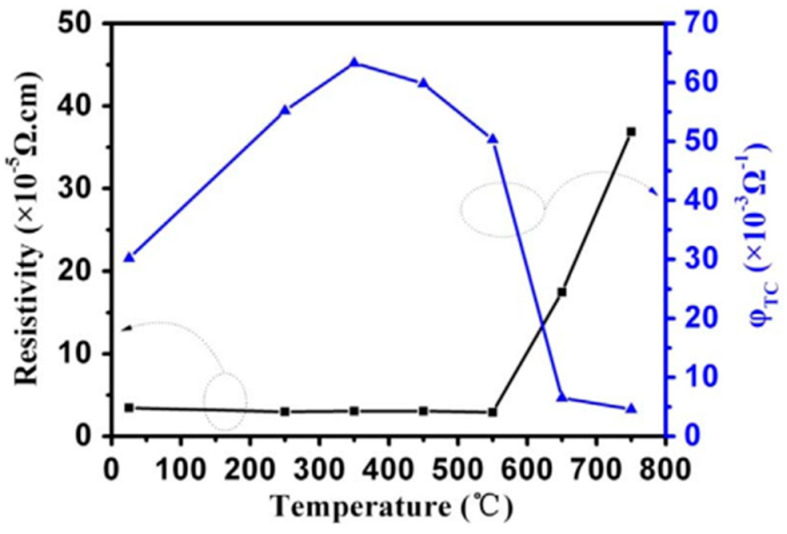
Resistivity and FOM values of AZO/Ag/AZO films for the as-deposited sample and different annealing temperatures. Reproduced from [55] with permission from the copyright clearance center.

Figure 16 illustrates how the figure of merit (FOM) value and resistivity vary with the thermal treatment temperature. Because of better crystal quality during thermal annealing, the resistivity of the AZO/Ag/AZO multilayer films decreased marginally as the annealing temperature rose to 550 °C. Nevertheless, the AZO/Ag/AZO multilayer films’ resistivity increases drastically when the annealing temperature climbs over 550 °C [55]. Additionally, it is discovered that the AZO/Ag/AZO multilayer films’ FOM rises initially and subsequently drops with increasing temperature, reaching the maximum at 350 °C in a vacuum atmosphere of 63.3 × 10^−3^ Ω^−1^. Because of much higher sheet resistance, the FOM drops when the thermal treatment temperature rises above 550 °C. The resistivity was found to be 3.7 × 10^−4^ Ω cm, even after high-temperature annealing up to 750 °C, relative to 1 × 10^−3^ Ω cm, which fulfills the practical need for transparent electrodes as well. The results indicate that some devices made using the AZO/Ag/AZO multilayers coated on mica may be used at high temperatures. Furthermore, there was no discernible change in the multilayers’ sheet resistance following a severe bending test. These findings suggest that AZO/Ag/AZO multilayer films were grown on mica, which is flexible and resistant to high temperatures, and may find use in next-generation flexible optoelectronics. At room temperature, each measurement was completed.

## 6. Applications of Transparent Conducting Thin Films and Coatings

There are several applications for TCFs. TCOs are particularly well suited for use in optoelectronic devices, including OLEDs, field effect transistors, solar cells, electrochromic devices, touchscreens, and sensors, because of their exceptional thermal and chemical stabilities, low contact resistance, and high stretchability [56]. Energy-conserving windows are made by making use of their propensity to reflect thermal infrared heat [57]. These low emissivity (also known as “low-E”) windows currently house the majority of TCFs’ applications. The selection of the spectrum offered by free electrons is the foundation of low-emissivity coatings. When the wavelength of the TCF exceeds that of the plasma, it reflects energy and is transparent at shorter wavelengths. Shorter-wavelength visible light is transmitted, while heat (infrared) waves are reflected because the plasma wavelength is frequently in the infrared. TCFs are used in oven windows in order to keep the exterior temperature at a level that is safe to touch, which leads to energy savings [57]. Flat panel displays (FPDs) and solar cells’ front-surface electrodes utilize TCFs’ electrical conductivity. Cars with auto-dimming rearview mirrors and electrically operated “smart” windows have two kinds of TCFs with an electrochromic material as a sandwich layer. To keep freezer display cases defrosted and to defrost windows in cars, an electric current is run via TCFs. TCFs remove the static charge from xerographic copiers’ windows. TCF layers are etched to create glass touchscreen control panels. Moreover, TCFs can be shaped into transparent radio antennae integrated into car windows, invisible security circuits on windows, and transparent electromagnetic shields. As a result, certain TCFs work best in various applications. We shall now examine how these requirements relate to several TCF applications.

### 6.1. Flat Panel Displays (FPDs)

As the front electrode, TCFs are diversified when it comes to FPD types. When creating patterns in the TCF electrode, etchability is a crucial factor to consider [33]. ITO is more commonly used than tin oxide because it is easier to etch. Tin oxide is harder to etch. The low ITO deposition temperature is another issue for color displays, where TCFs are placed over heat-sensitive organic dyes [56]. ITO is preferred in highly patterned displays because of its low resistance. This is because the ITO layer may be deposited extremely thinly, maintaining a relatively smooth etched topography. ZnO is less expensive and simpler to etch. LEDs can be made with ZnO-based semiconductors; however, ITO needs the development of materials with high conductivity and transparency to be employed as front electrodes. For flat panel displays, ITO is still preferred, while FTO is the most commonly used TCF [33].

### 6.2. Touchscreen Panel Controls

Etched TCFs on glass are used to create touchscreen panels, such as those on ATM screens, controls of elevators, and screens for appliances. They detect the presence of a finger by capacitively passing through the glass or by direct contact. TCFs are a crucial part of solar and touchscreen technologies [58]. The most widely utilized material for TCFs is ITO or In_2_O_3_:Sn [58]. TiO_2_ is a feasible option for these applications because of its affordability and durability [19]. Because of their ability to multitask and perform many touch functions, TSPs that are the capacitive type are currently being employed in place of traditional TSPs that are the resistive type [59]. The mechanical resilience of the flexible TCO electrode to substrate bending should not cause any modifications to its optical or electrical characteristics. To assist engineers in accurately regulating the ITO bar production process and generating the appropriate touch panel with the required film thickness and resistance, an intelligent control system has been devised [60]. It is essential that the TCF be as thin as possible in specific applications. For instance, in high-resolution screens, the device’s height varies because of the TCF’s required etched patterns. The electrical resistivity is a crucial material property that should be kept as low as possible to maintain the smoothest topography possible; some common values are provided in Table 5 [33].

As Table 5 illustrates, the highest achievable plasma frequency of TCFs and the electron concentration often rise in the same order as the resistivity [33]. For ZnO:F, the associated plasma wavelengths reduce to around 2 μm, whereas for TiN, they reach 0.7 μm (red light), as shown in the table.

### 6.3. Solar Cells

One form of photovoltaic technology that converts photon energy directly to electrical power is the solar cell [1,57]. Transparent electrodes are placed on the front faces of solar cells. In single-crystal silicon cells, the front electrode is a highly doped layer of silicon [33]. In thin-film cells, a TCF layer serves as the front electrode. Certain amorphous silicon solar cells and cadmium telluride are manufactured on a glass superstrate covered with SnO_2_:F. The main considerations in this decision are affordability and thermal stability. The p-type amorphous Si layer can be electrically connected at a low resistance thanks to the maximum working function of SnO_2_:F. On flexible steel or plastic substrates, other amorphous Si cells are grown; in this instance, the topmost TCF needs to be deposited at a reasonably low temperature on thermally delicate cells. This application is selected for ZnO or ITO. The reason is that they both can be satisfactorily deposited at low temperatures (usually <200 °C).

### 6.4. Buildings with Low-Emissivity Windows

Because the free electrons in glass reflect infrared light at longer wavelengths than the plasma wavelength, TCFs on window glass increase the energy efficiency of the window [33]. It has a similar effect as a thermos bottle’s silver coating. Because the plasma black-body wavelength radiation in cold climates is somewhat long, roughly 2 μm, the majority of the sun’s spectrum is intended to be converted to heat inside the building. The optimal material for this application is F-doped tin oxide because it combines cost efficiency, good durability, and an appropriate plasma wavelength. Worldwide, buildings have billions of square feet of TCF-coated window glass installed. In warm regions, it is preferable to obstruct a short black-body radiation wavelength of less than 1 μm in order to allow for the building to reflect the near-infrared part of the incident sunlight outside. For this use, titanium nitride and silver have short-enough plasma wavelengths. Despite its short lifespan, silver is frequently employed for this purpose; it is encased behind double-glazed panes to keep out moisture and air. Much more resilient, titanium nitride can be applied to exposed surfaces, even single-glazed windows. Although TiN-coated glass is often found on large commercial buildings, its reflective gold tint is not common for home windows.

A TCF’s optical properties are split by the conduction electrons’ plasma frequency [33]. The material acts as a transparent dielectric because the electrons cannot react at frequencies higher than the plasma frequency. At frequencies lower than the plasma frequency, incoming radiation on the TCF is reflected and absorbed. Most TCF materials fall into the upper transparent frequency range for the visible region, while the near-infrared section of the spectrum corresponds to the plasma frequency. There is a rough correlation between the square root of the conduction electron concentration and the plasma frequency [33].

### 6.5. Transistors

An apparatus that amplifies or switches electrical power and electronic impulses is called a transistor. A transistor can amplify a signal by maximizing the regulated output in relation to the input power. It is also a necessary part of modern technical apparatus. Si or Ge are the usual materials used to make semiconductor devices. A thin-film transistor (TFT) is created by shaping the active semiconductor layer into a thin film. Transistors with a transparent semiconductor thin film and transparent electrodes (such as ITO) are known as transparent thin-film transistors (TTFTs). The primary difficulty with these devices is that the transparent thin film’s deposition temperature needs to match that of traditional substrates [1]. To create a thin layer that is both electrically and optically equivalent to ITO while preserving the substrate, a high-temperature deposition method must be used instead of ITO. In thermoelectric thin-film transistors, metal nanoparticles have shown great promise as a source and a drain, allowing for deposition on a range of substrates, including flexible and stiff ones, and greatly lowering production costs. Furthermore, these nanomaterials combined with polymers (composites) provide a feasible substitute for costly and brittle traditional transparent conductive metal oxides (such ITO) [1].

### 6.6. Electromagnetic (EM) Shielding

The unrestricted flow of electromagnetic radiation through the atmosphere has the potential to disrupt or impede the functioning of electrical circuits. Electromagnetic radiation from an external source is usually the cause of EMI. When building a circuit, it is critical to keep environmental electromagnetic radiation (EMI) to a minimum and to include enough electromagnetic shielding to withstand it. In EM shielding, conductive or magnetic materials are employed to block electromagnetic radiation in order to reduce EMI. EM shielding can be used to protect a circuit from excessive electromagnetic radiation if the circuit design is unable to do so. The electrical conductivity of EM shielding materials has a major impact on how effective they are. Historically, EM shielding has been achieved by the use of metal sheets (Al, Cu, and steel). However, because of their good mechanical properties, low weight, and affordability, nanocomposites—polymer reinforced with highly conductive fillers—have gained prominence recently. High-aspect-ratio nanomaterials, including metal nanowires (NWs), preserve the mechanical properties of polymers while requiring very little filler loading, making the product commercially feasible [1]. It appears that electromagnetic waves traveling through windows can be used to intercept computer conversations and other types of communication [33]. TCs with low sheet resistance can block these stray impulses. For this aim, the finest materials are silver and ITO.

### 6.7. Dielectrics

Conductive metal nanoparticles can be used as conductive fillers in polymers, changing the polymers’ dielectric characteristics in the process. When a tiny quantity of conductive filler is added, the polymers’ dielectric constant will significantly rise [1]. In particular, polymers with very low percolation thresholds may have a considerable increase in their dielectric constant when exposed to metal NWs and other conductive nanomaterials with high aspect ratios. The plasma frequency of a TCF’s conduction electrons divides its optical characteristics [33]. The material acts as a transparent dielectric at frequencies greater than the plasma frequency because electrons are unable to react at these ranges. The TCF absorbs and reflects incident radiation at frequencies that are lower than the plasma frequency.

### 6.8. Aerospace Industry

Lightweight composites with conductive metal nanoparticles, as fillers in polymer matrices, with frequencies lower than the plasma frequency have attracted a lot of interest from the aerospace sector [1]. It is necessary to replace heavy-metal meshes, like Cu mesh, that shield an aircraft’s exterior from lightning strikes by creating a conductive channel, with lighter conductive materials that have qualities that are on par with or better than ones made with conventional materials. To create new materials as an alternative to those now used in aircrafts, a number of issues must be resolved, such as low weight, high conductivity, environmental friendliness, flexible manufacturing, high mechanical strength, and ease of maintenance.

### 6.9. Medical Applications

One of the limitations of applications in electronic implants is the differentiation between living tissues and materials utilized in implants [1]. The majority of tissues are pliable and soft, but the electronic implant’s primary materials, mainly metal and semiconductors, are brittle. Bone support is provided via long-lasting implants. Sensors and their insertion in soft tissues are appropriate uses for flexible materials [1]. Thus, depending on the reason for the implantation, a different implant type must be chosen for each circumstance. Stretchability and flexibility are important considerations when selecting an appropriate electrical implant. Bending and stretching of the electronic implant results in the formation of cracks that isolate electrical pathways and lead to failure. It might be possible to keep the material conductive by bridging the fractures under stretching with conductive materials with high aspect ratios, such as metal NWs.

### 6.10. Lithium-Ion Batteries (LIBs)

Lithium ions in LIBs move from the negative electrode to the positive electrode during discharging and charging [1]. LIBs are a kind of rechargeable battery. In terms of portable electronics and rechargeable batteries for homes, LIBs are among the most widely used varieties. Lithium-ion batteries (LIBs) are becoming more and more popular for electric vehicles and aerospace and military applications because of their minimal self-discharge and high energy density. One of the advantages of using LIBs in new applications is that they can be used to replace conventional heavy-metal current collectors with novel materials that will efficiently lower the weight of the final product [1]. As conductive thin films infill for a very conductive current collector used in LIBs’ anodes and cathodes, it has been claimed that conductive metal nanoparticles can be employed. These conductive metal nanoparticles improve the mechanical properties of LIBs and make LIBs lighter.

### 6.11. Defrosting Windows

Supermarket freezers use electric current to run through TCFs on their display glass to keep moisture from condensing and obstructing the view [33]. Tin oxide was selected for this purpose primarily because of its high durability and low cost. In older times, during WWII, the use of TCFs was to defrost airplane glass, which allowed for high-altitude bombing. Until after the war, the finding of TCFs was maintained as a secret. In contemporary cockpits, ITO has taken the position of tin oxide because of its reduced resistance, which allows for the defrosting of bigger window regions at a relatively low voltage (24 V).

Cars with auto-dimming rearview mirrors and electrically operated “smart” windows have two TCFs with an electrochromic (EC) material in between. To maintain freezer display cases frost free and to defrost windows in cars, electric current is run via TCFs [33]. The metal layers in the windshield are shielded by a layer of laminate between two glass sheets. For instance, as the thickness of the layer increases, it results in a higher electrical conductivity but also a higher transparency. The time it takes to defrost the windshield is reduced by having the lowest possible electrical resistance (for a fixed maximum available power).

### 6.12. Circuits

Nearly all electronic and optoelectronic devices entirely comprise electrical circuits, which find applications in smart labels, RF identification tags, wearable electronics, transistors, LEDs, and LCDs [1]. Because these materials may be printed as conducting thin films on a variety of substrates (rigid or flexible) using simple deposition processes, the ability to generate conductive inks utilizing conductive nanoparticles was a significant success. Creating conductive inks reduced the cost of creating electrical circuits significantly because these advanced inks do not require costly substrates, vacuum processing, or traditional lithography. TCF-coated glass can be used to protect priceless art pieces or utilized as a component of invisible security circuits for windows [33]. Moreover, TCFs offer some protection against fading caused by UV rays. Any TCF might be used, with the exception of colored TiN. Multilayers of silver/ZnO offer the best UV protection.

### 6.13. Durability of Glasses

Certain tin oxide coatings have nothing to do with the material’s electrical conductivity and are just intended to capitalize on tin oxide’s exceptional durability [33]. Barcode readers’ panes are coated with tin oxide to increase their resistance to abrasion. Hydrofluoric acid does not harm tin oxide; however, it does wash glass. Etching kits with hydrogen fluoride (HF) are intended to etch identification marks on automobile glass, but vandals have used them to etch inscriptions on windows. Tin oxide coatings protect windows from these kinds of attacks. Cadmium stannate requires very refractory substrates (especially high-melting-temperature glass) to produce its finest details, while ITO is commonly selected for deposition on substrates that are sensitive to heat, such as plastics [33].

### 6.14. Oven Windows

Tin-oxide-coated oven windows reduce the external glass’s temperature to a reasonable level, making it safer [33]. This allows for windows to be used at extremely high temperatures in self-cleaning ovens. The ovens’ energy efficiency is further enhanced by the tin oxide covering. High-temperature stability, chemical and mechanical endurances, and affordability are the primary factors that led to this material’s selection. Certain transparent lab ovens are made totally of (TCF)-coated glass, which also works as the oven’s electrical resistance.

## 7. Stability of AZO Thin Films

Transparent conducting AZO thin films have exhibited higher conductivities in oxidizing atmospheres at high temperatures and high etching rates in alkaline and acidic solutions, indicating that they are less stable chemically and electrically than undoped ITO thin films and In_2_O_3_ [61,62,63,64,65]. Additionally, when conducted in a setting with higher humidity (air at 60 °C and 90% relative humidity), it has been noted that the resistivities of transparent conducting AZO thin films grown on glass substrates at temperatures below 200 °C, with thicknesses ranging from 20 to 200 nm, increase with increasing exposure time [61,63,64]. Transparent electrodes for AR coatings must, nevertheless, be durable when utilized in various environmental conditions. However, their use in AR coatings must be robust in an environment with a relatively high humidity. It has been discovered that increasing the crystallinity and regulating the chemical composition through co-doping with another impurity and adjusting the kind and quantity of the doped impurity can both increase the stability of AZO thin films [63,64]. The ALD of AZO films on flexible substrates (transparent and yellow Kapton) preserves excellent thermoelectric properties (generator applications) with excellent stability in the presence of humidity and bending [66]. Thus, it can be said that RF and DC magnetron sputtering and ALD deposition are appropriate for the realistic development of AZO thin-film electrodes for LCDs, regardless of whether hydrogen gas is used [49].

**Figure 17:**
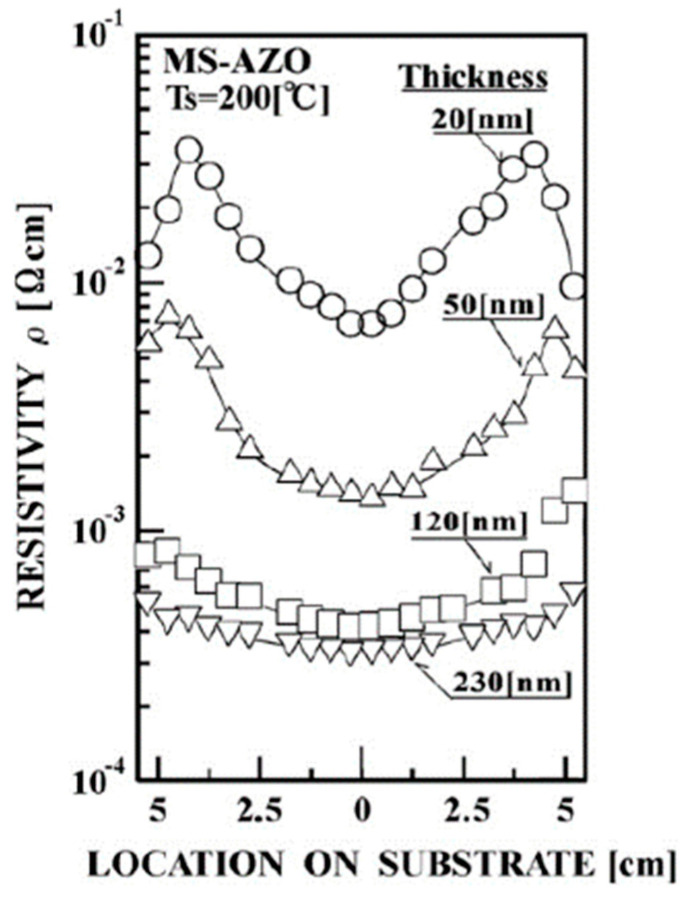
Resistivity spatial distribution as a function of the film thickness for AZO thin films produced by RF-DC with H2 injection. Reproduced from [49] with permission from the copyright clearance center.

Figure 17 displays the ρ spatial distribution as a function of the film thickness for AZO thin films that are deposited using rf + dc − MS at 200 °C on glass substrates while hydrogen gas is introduced [49]. Although an AZO thin film with a thickness of 200 nm produced a fairly uniform resistivity distribution, as the thickness was decreased below roughly 100 nm, the resistivity obtained increased drastically, and the resistivity’s spatial distribution degraded. However, it should be noted that all these thin films were produced with deposition parameters of 30 W of DC power, 70 W of RF power, and a 2% H_2_ gas partial pressure, which were intended for 200 nm thick films. It was found that the pre-sputtering settings and target characteristics of the early stage of the deposition might regulate the resistivity’s thickness dependency and distribution. Specifically, it is critical that AZO thin films be made with target qualities that can be controlled, including a low oxygen content that can be adequately decreased by MS deposition [49].

**Figure 18:**
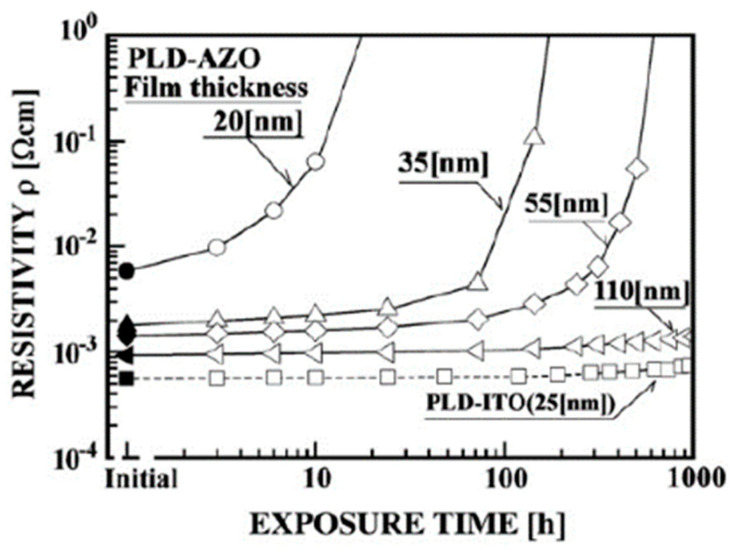
Resistivities of AZO films, made with different thicknesses via PLD, as a function of the exposure duration. Reproduced from [49] with permission from the copyright clearance center.

Figure 18 illustrates an example of the resistivities of AZO films made using PLD, at different thicknesses (20, 35, 50, and 100 nm), as a function of the exposure duration. At a temperature of 140 °C, these AZO thin films were produced on glass substrates with an Al content of around 8 at.% [64,65]. It is crucial to keep in mind that PLD-AZO thin films thicker than 100 nm are stable enough to be applied to transparent electrodes in practical settings, whereas films thinner than 50 nm might not be suitable. On the other hand, even at a thickness of 25 nm, an ITO thin film produced under the same conditions remained rather stable [49].

However, the distinction between ITO and AZO can be described by the presence of an O_2_-adsorbing site that is related to either the covalent or ionic form of hanging bonds. Consequently, thin films thinner than around 50 nm, including polycrystalline AZO, are unstable. Specifically, it appears likely that practical transparent electrode applications will have difficulty achieving a degree of stability similar to that of AZO.

## 8. Conclusions

Because of their superior electrical and chemical stabilities over those of AZO, it can be said that In_2_O_3_ and ITO thin films are the best replacement materials for AR coating applications. ITO is the optimal option when taking the environment and resource availability into account. This review paper highlights the primary issues surrounding the alternative of AZO for ITO film applications used in AR coatings. Specifically, the best deposition technique for preparing realistic TC ITO thin films is (rf + dc − MS) deposition, which can be achieved with or without the introduction of H_2_ gas. The primary concern with integrating this technology into the many realistic AR-coated fabrication lines will remain at focus. It is likely to remain challenging, though, to achieve an ITO-like stability level for impurity-doped AZO coatings that are less than 30 nm thick. It is crucial to note that the biggest issues with replacing ITO with AZO are those related to the supply and the environment.

## Figures and Tables

**Table 1:** Choice of TCOs considering various properties [33].

Property	Material
Lowest price	SnO_2_:F
Least harmful	SnO_2_:F; ZnO:F
Optimal resistance to H plasmas	ZnO:F
Maximum frequency of plasma	In_2_O_3_:Sn; TiN; Ag
Minimum frequency of plasma	ZnO:F; SnO_2_:F
Maximum conductivity	In_2_O_3_:Sn
Maximum transparency	Cd_2_SnO_4_; ZnO:F
Maximum work function; best contact to *p*-Si	ZnSnO_3_; SnO_2_:F
Minimum work function; best contact to *n*-Si	ZnO:F
Optimal thermal stability	Cd_2_SnO_4_; SnO_2_:F; TiN
Optimal chemical durability	SnO_2_:F
Optimal mechanical durability	SnO_2_:F; TiN
Minimum deposition temperature	ZnO:B; In_2_O_3_:Sn; Ag
Easily etched	TiN; ZnO:F

**Table 2:** Materials with associated dopants [32].

Material	Dopant	Resistivity Value
ITO	Ta; Nb; W; Zr; Ti; Mo; Ge; Sn	Excellent
SrTiO_3_	La; Nb	Bad
GaInO_3_	Sn; Ge	Average
CdO	In; Sn	Excellent
ZnO	Zr; Ti; Ge; Si; In; B; Ga; Al	Excellent
CdIn_2_O_4_	CdO–In_2_O_3_ system	Good
Zn_2_SnO_4_	ZnO–SnO_2_ system	Average

**Table 3:** Various O_2_ flow rates with properties of ITO thin films [42].

Flow Rate of Oxygen (sccm)	Sheet Resistance (Ω/sq.)	Resistivity (10^−4^ Ω cm)	Transmittance (%)	Root-Mean-Square Surface Roughness) RMSSR (nm)	Bandgap (eV)	Figure of Merit (10^−4^ Ω^−1^)
8	130	15.6	75	0.52	4.15	4.33
10	74	8.9	82	0.74	4.17	18.57
12	60	7.2	84	0.93	4.19	29.15
14	84	10.1	80	0.38	4.16	12.78

**Table 4:** Electrical characteristics of the PLD-deposited ITO films, estimated from the Hall measurements [48].

Samples	Sheet Resistance (Ω/sq.)	Carrier Concentration (cm^−3^)	Mobility (cm^2^/Vs)	Resistivity (Ω·cm)
ITO/glass	5.3	3.3 × 10^21^	10.6	1.8 × 10^−4^
ITO/NP–glass	8	1.5 × 10^21^	15.1	2.8 × 10^−4^

**Table 5:** Approximate plasma wavelengths minimum resistivities for certain TCFs [33].

Material	Plasma Wavelength	Resistivity
ZnO:F	>2.0	400
SnO_2_:F	>1.6	200
ZnO:Al	>1.3	150
Cd_2_SnO_4_	>1.3	130
In_2_O_3_:Sn	>1.0	100
TiN	0.7	80
Ag	0.4	1.6
